# Supplementary material for: MPP8 is essential for sustaining self-renewal of ground-state pluripotent stem cells
Source: Nat Commun. 2021 May 24;12:3034. doi: 10.1038/s41467-021-23308-4 (PMC8144423; doi:10.1038/s41467-021-23308-4)
Supplement: Supplementary file 1 — Supplmentary Information [file 41467_2021_23308_MOESM1_ESM.pdf]

Supplementary Information

MPP8 is essential for sustaining self-renewal of ground-state pluripotent stem cells

Müller et al.

This PDF file includes:

- Supplementary Figures 1-12
- Supplementary Tables 1-10

Other supplementary information not included in this file:

- Supplementary Data 1 (separate .xlsx file): sgRNA sequences, raw counts of sgRNAs from the epi library CRISPR/Cas9 screen and identified hits
- Supplementary Data 2 (separate .xlsx file): Differentially regulated transcripts obtained from RNA-seq
- Supplementary Data 3 (separate .xlsx file): Protein abundances obtained from mass spectrometry using Proteome Discoverer software and statistical analysis performed using Perseus software
- Supplementary Data 4 (separate .xlsx file): Genomic coordinates and gene annotations of MPP8 binding sites

Supplementary Fig. 1

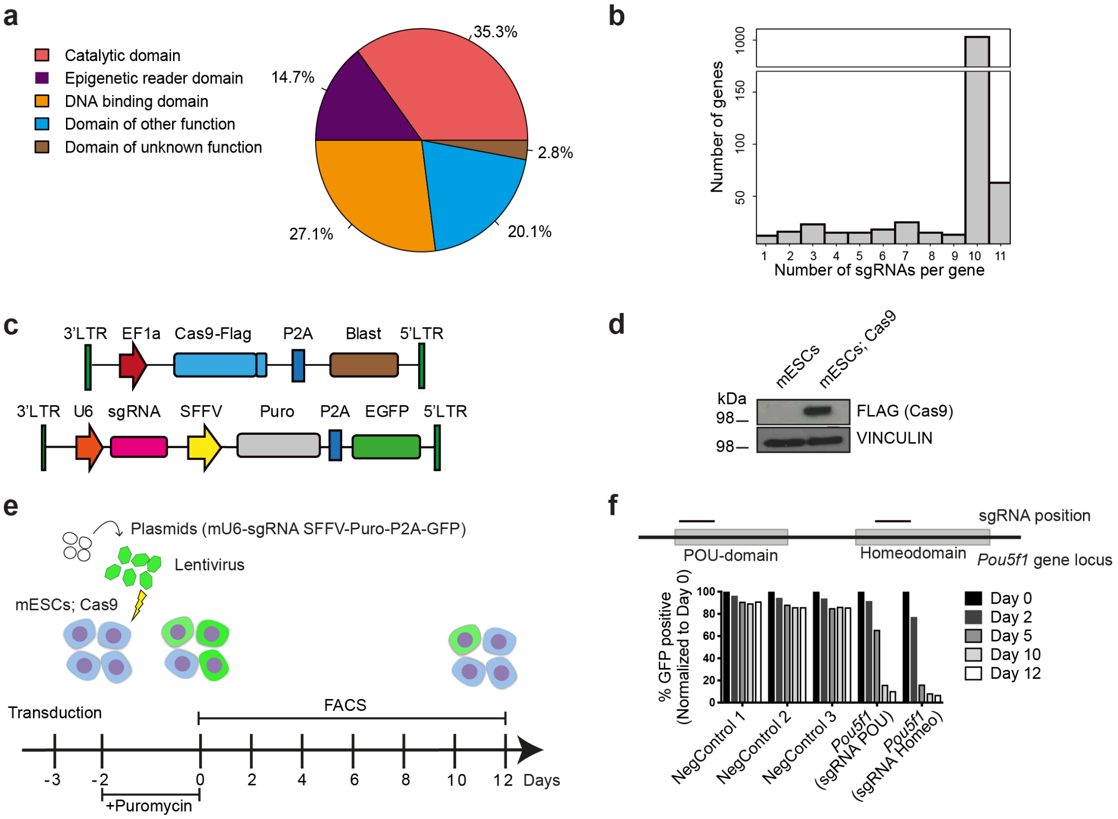

Supplementary Figure 1. Generation of a sgRNA library targeting functional domains of potential epigenetic factors. a Pie chart showing the representation of different protein domain categories in the CRISPR/Cas9 screening library. Domains were ranked into five categories according to their targeting relevance. **b** Distribution of the number of sgRNAs per gene in the epi library. **c** Schematic map of the lentiviral constructs used for CRISPR/Cas9 screening. **d** Cas9 protein expression in mESCs after transduction with a lentiviral Cas9 construct, and VINCULIN as loading control (*n* = 1). **e** Experimental strategy of the competition-based proliferation assay for evaluation of the functionality of CRISPR/Cas9-mediated targeting of protein domains in mESCs. GFP+ cells are indicated in green. **f** Competition-based proliferation assay targeting functional domains in *Pou5f1* (encoding OCT4) or using non-targeting control sgRNAs (NegControl 1-3) in Cas9-expressing mESCs. Graphs show the proportion of GFP+ cells relative to day 0 (*n* = 1). Source data are provided as a Source Data file.

Supplementary Fig. 2

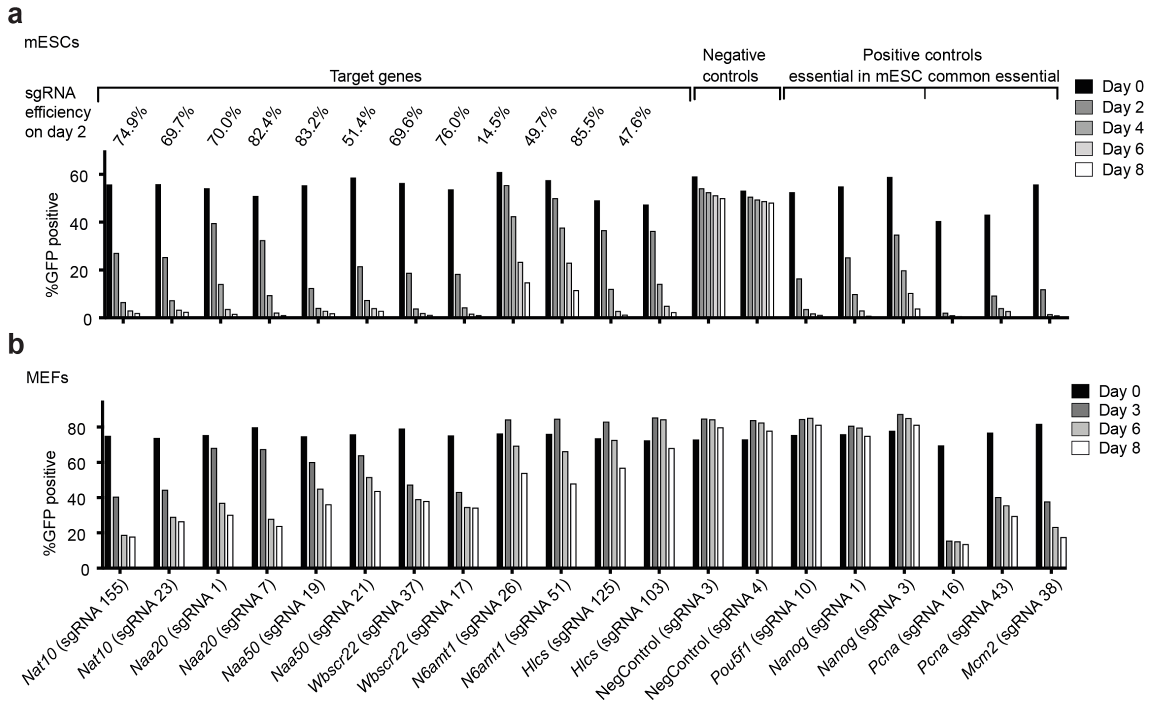

Supplementary Figure 2. Validation of identified hits in mESCs and MEFs. a, b Competition-based proliferation assays in indicated Cas9-expressing cell lines. GFP expression was monitored over a time course of eight days. sgRNAs targeting the common essential genes *Pcna* and *Mcm2* served as positive control for mESCs and MEFs, while sgRNAs targeting the mESC-specific genes *Nanog* and *Pou5f1* (encoding OCT4) served as positive controls in mESCs and negative controls in MEFs. Non-targeting (NegControl) sgRNAs served as negative controls in both cell lines. Two independent sgRNAs were directed against each gene of interest: *Nat10*, *Naa20*, *Naa50*, *Wbscr22*, *N6amt1* and *Hlcs* (n = 1). mESCs = mouse embryonic stem cells; MEFs = mouse embryonic fibroblasts. Source data are provided as a Source Data file.

Supplementary Fig. 3

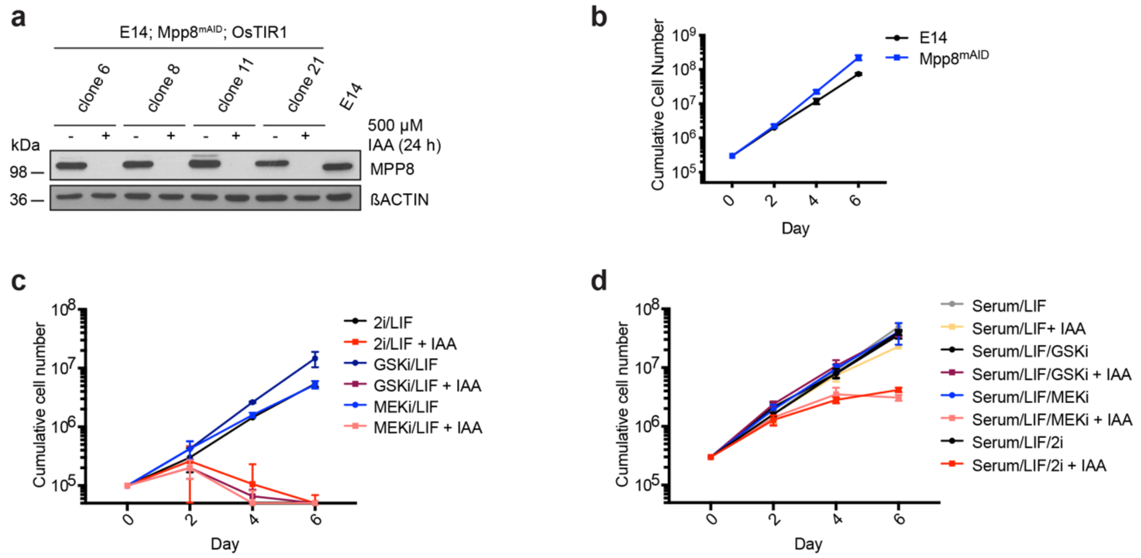

Supplementary Figure 3. Proliferation changes of mESCs grown under distinct culture conditions in response to MPP8 depletion. a Establishment of the auxin-induced depletion system via homozygous tagging of the genomic *Mphosph8* locus with the C-terminal miniAID (mAID) sequence and ectopic expression of OsTIR1. Assessment of the established system using MPP8 and βACTIN (loading control) expression levels in four different clones ± 500 μM IAA for 24 hours (*n* = 1). **b** Cell proliferation assay of Mpp8^{mAID} or E14 (control) cells grown in serum/LIF culture (mean ± s.d., *n* = 3 independent experiments). **c, d** Cell proliferation assays of Mpp8^{mAID}; OsTIR1 cells grown in 2i/LIF, MEKi/LIF and GSKi/LIF culture conditions (**c**) or in serum/LIF conditions with addition of no inhibitor, MEK inhibitor, GSK inhibitor or both (**d**), ± 500 μM IAA (mean ± s.d., *n* = 3 independent experiments). Source data are provided as a Source Data file.

Supplementary Fig. 4

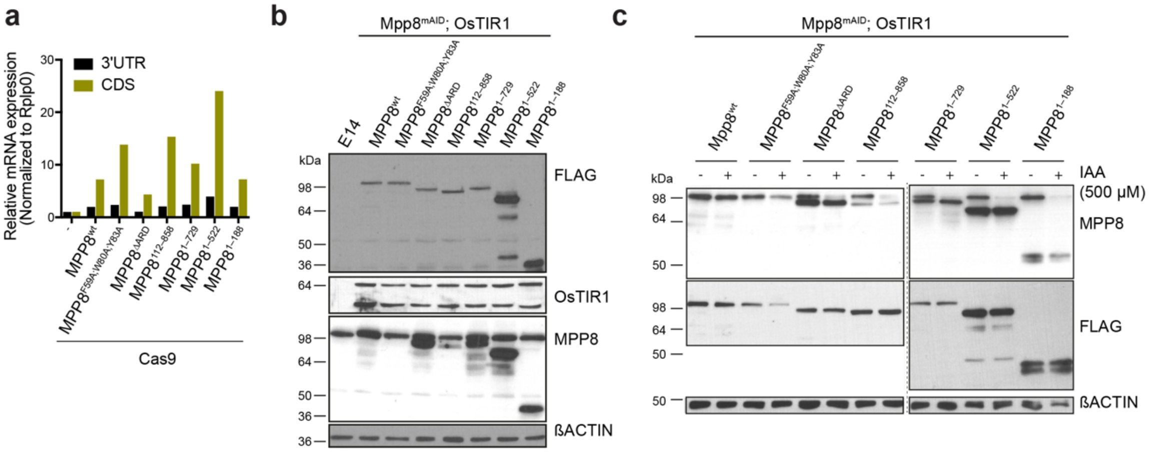

Supplementary Figure 4. Expression levels of MPP8 fragments. a qRT-PCR analysis of endogenous *Mphosph8* (3'-UTR) or total *Mphosph8* (coding sequence, CDS) in mESCs stably expressing wildtype MPP8 or the indicated MPP8 mutants as well as Cas9 (*n* = 1). **b** Western blot analysis of FLAG, MPP8, OsTIR1 and βACTIN (loading control) in control (empty; E14) cells or Mpp8^{mAID} cells stably expressing MPP8 wildtype or the indicated MPP8 mutant proteins as well as E3 ligase OsTIR1 (*n* = 1). **c** Western blot analysis of FLAG, MPP8 and βACTIN (loading control) in Mpp8^{mAID} cells stably expressing MPP8 wildtype or the indicated MPP8 proteins as well as E3 ligase OsTIR1 ± 500 μM IAA for 20 hours (*n* = 1). Source data are provided as a Source Data file.

Supplementary Fig. 5

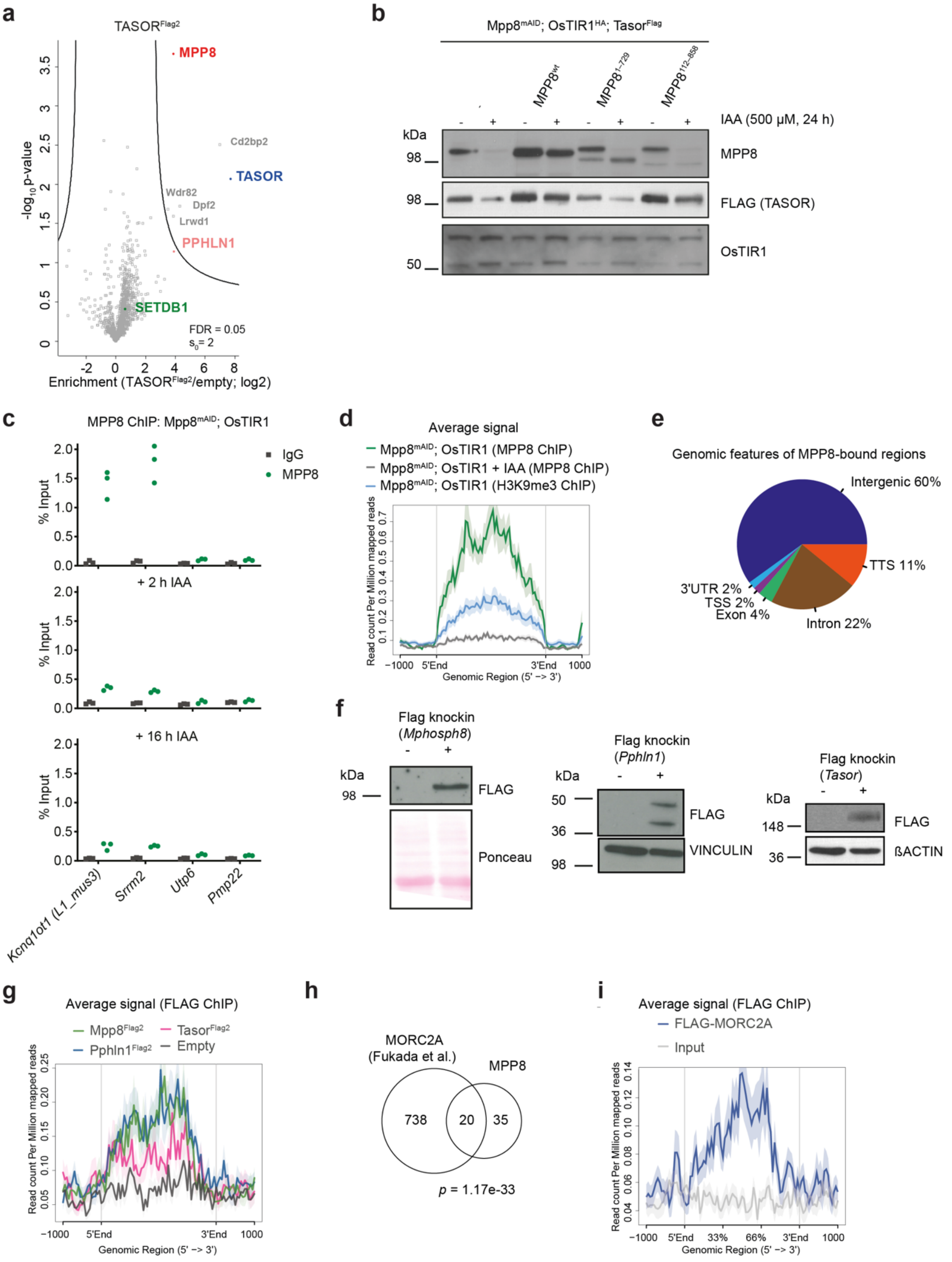

Supplementary Figure 5. The HUSH core complex assembles and co-localizes at MPP8 binding sites in mESCs. a LC-MS/MS of FLAG pull-downs in Tasor^{Flag2} mESCs. Parental untagged mESCs serve as background control. HUSH complex members are color-highlighted. Statistical analysis was performed using Perseus (see Methods, *n* = 2 biologically independent samples). **b** Western blot analysis of MPP8, FLAG (endogenous TASOR) and OsTIR1 in Mpp8^{mAID}; OsTIR1; Tasor^{Flag2} cells and cells additionally expressing (non-tagged versions of) MPP8^{wt}, MPP8$^{1–729}$ and MPP8$^{112–858}$, respectively, ± 500 µM IAA for 24 hours (*n* = 1). **c** ChIP-qPCR data probing kinetics of MPP8 depletion on two known MPP8 target (*L1_mus3*, *Kcnq1ot1*; *Srrm2*) and non-target (*Utp6, Pmp22*) loci in untreated Mpp8^{mAID}; OsTIR1 cells or upon addition of auxin for 2 hours and 16 hours, respectively. ChIP was performed using an antibody against MPP8 or IgG (control) (*n* = 3 technical replicates). **d** Aggregate plot comparing the average MPP8 ChIP signal over high confidence peaks (*n* = 55) in Mpp8^{mAID}; OsTIR1 cells ± 500 µM IAA for 16 hours and the average H3K9me3 ChIP signal over high confidence peaks (*n* = 55) in Mpp8^{mAID}; OsTIR1 cells. **e** Genomic features of high confidence (*n* = 55) MPP8-bound regions. The genomic annotation of the region occupied by the center of the peak was determined. TSS = Transcription start site (-1 kb to +100 bp), TTS = Transcription termination site (-100 bp to +1 kb). **f** Western blot analysis of FLAG or loading control (Ponceau staining, VINCULIN or ßACTIN) in empty parental or endogenously FLAG-tagged *Mphosph8*, *Pphln1* and *Tasor* loci in mESCs (*n* = 1). **g** Aggregate plot comparing the average FLAG ChIP signal over high confidence MPP8 peaks (*n* = 55) in mESCs harboring an endogenous C-terminal Flag signal in the genomic *Mphosph8*, *Pphln1* and *Tasor* loci, respectively. Parental untagged mESCs served as background control. **h** Overlap of binding sites for MORC2A (data published by Fukada et al., 2018) and MPP8 in mESCs grown in Serum/LIF and 2i/LIF culture conditions, respectively. *p* = 1.17e-33 (two-tailed Fisher's Exact Test). **i**) Aggregate plot comparing the average FLAG ChIP signal over high confidence MPP8 peaks (*n* = 55) in mESCs overexpressing FLAG-MORC2A grown in serum/LIF culture. Input served as control (data published by Fukada et al., 2018). Source data are provided as a Source Data file.

Supplementary Fig. 6

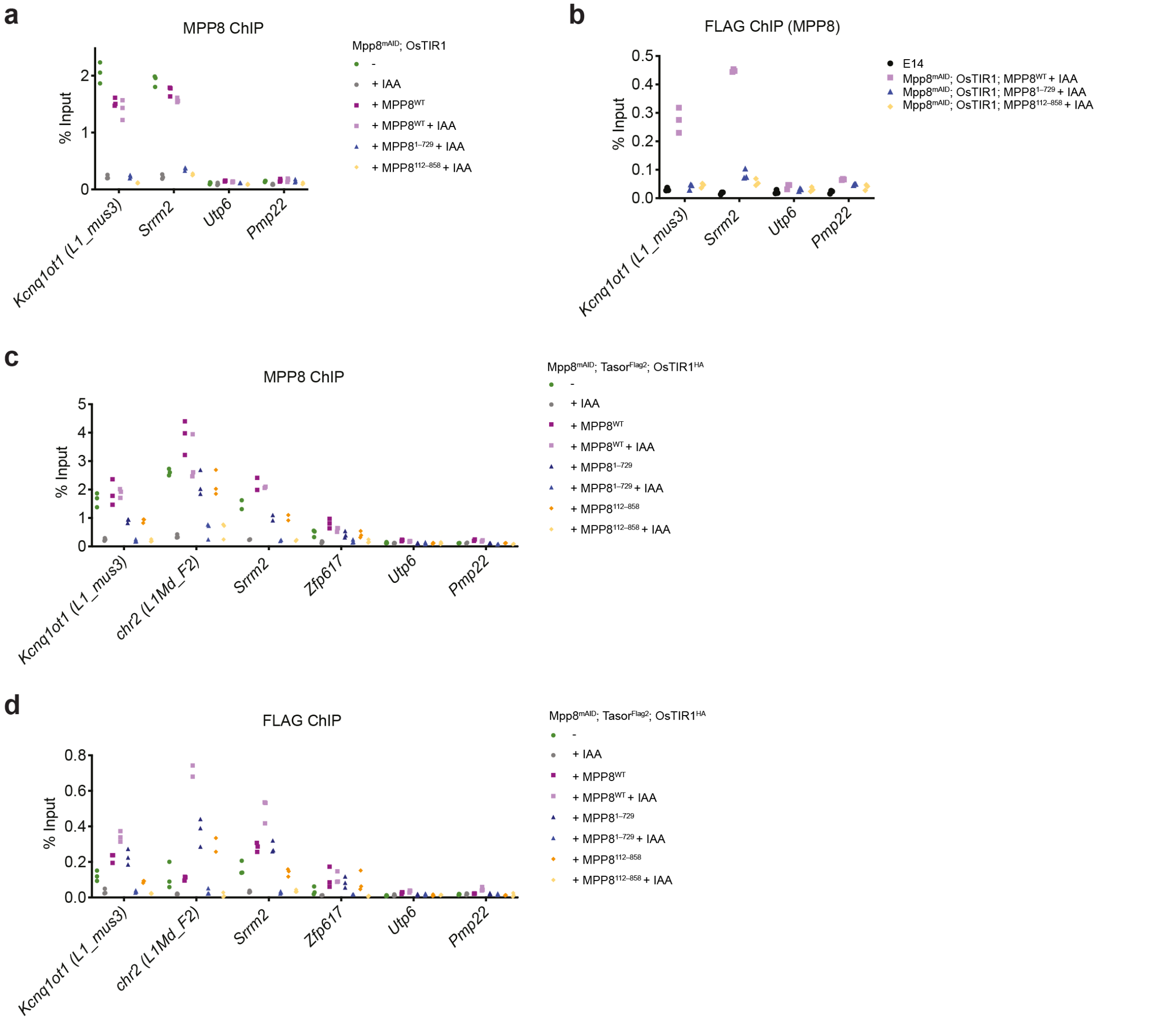

Supplementary Figure 6. TASOR is not associated with chromatin in the absence of the chromodomain of MPP8. a ChIP-qPCR validation of ChIP-seq data shown in Fig. 4g at two known MPP8 target loci (*L1_mus3*, *Kcnq1ot1*; *Srrm2*) and non-target (*Utp6*, *Pmp22*) loci in Mpp8^{mAID}; OsTIR1 cells or Mpp8^{mAID}; OsTIR1, MPP8^{wt} cells ± 500 µM IAA as well as Mpp8^{mAID}; OsTIR1 cells additionally expressing MPP8$^{1–729}$ and MPP8$^{112–858}$, respectively + 500 µM IAA for 16 hours (*n* = 3 technical replicates). **b** ChIP-qPCR validation of ChIP-seq data shown in (Fig. 4g) at two known MPP8 target (*L1_mus3*, *Kcnq1ot1*; *Srrm2*) and non-target (*Utp6*, *Pmp22*) loci in E14 cells (control) or Mpp8^{mAID}; OsTIR1 cells additionally expressing MPP8^{wt}, MPP8$^{1–729}$, MPP8$^{112–858}$, respectively, + 500 µM IAA for 16 hours (*n* = 3 technical replicates). **c** MPP8

ChIP-qPCR at two MPP8 binding sites that overlap LINE1 elements (*L1_mus3*, *Kcnq1ot1, L1Md_F2*, chr2), two non-LINE1 MPP8 binding sites (*Srrm2*, *Zfp617*) and two negative control loci not bound by MPP8 (*Utp6*, *Pmp22*) in Mpp8^{mAID}; Tasor^{Flag2}; OsTIR1^{HA} cells and cells additionally expressing (non-tagged versions of) MPP8^{wt}, MPP8$^{1–729}$ and MPP8$^{112–858}$, respectively, ± 500 μM IAA for 16 hours (n = 2 or 3 technical replicates). **d** FLAG ChIP-qPCR at two MPP8 binding sites that overlap LINE1 elements (*L1_mus3*, *Kcnq1ot1*; *L1Md_F2*, chr2), two non-LINE1 MPP8 binding sites (*Srrm2*, *Zfp617*) and two negative control loci not bound by MPP8 (*Utp6*, *Pmp22*) in Mpp8^{mAID}; Tasor^{Flag2}; OsTIR1^{HA} cells, and cells additionally expressing (non-tagged versions of) MPP8^{wt}, MPP8$^{1–729}$ and MPP8$^{112–858}$, respectively, ± 500 μM IAA for 16 hours (n = 2 or 3 technical replicates). Source data are provided as a Source Data file.

Supplementary Fig. 7

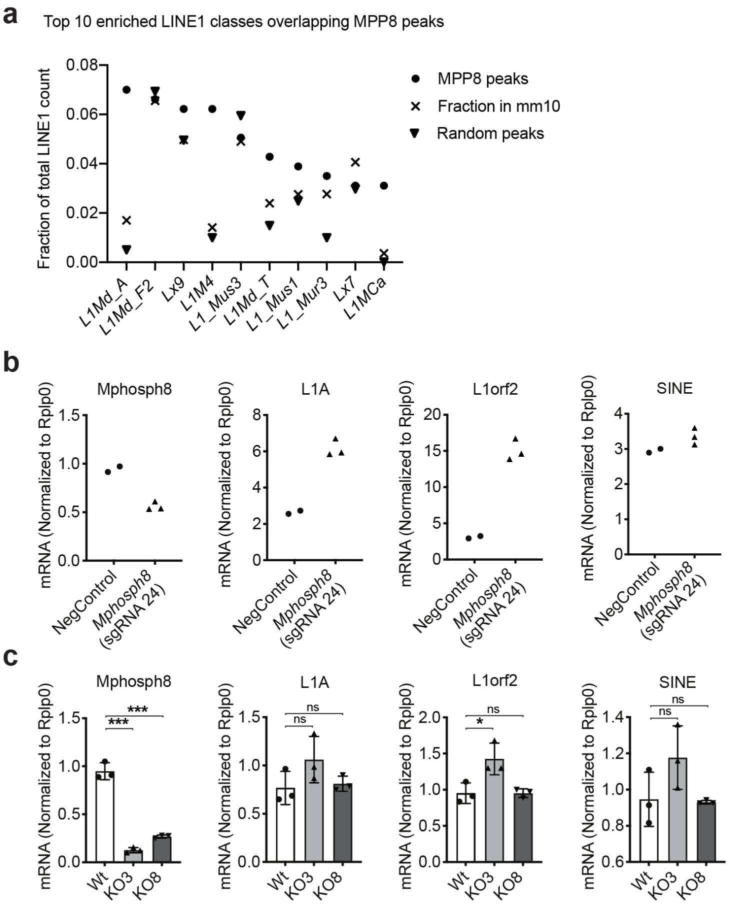

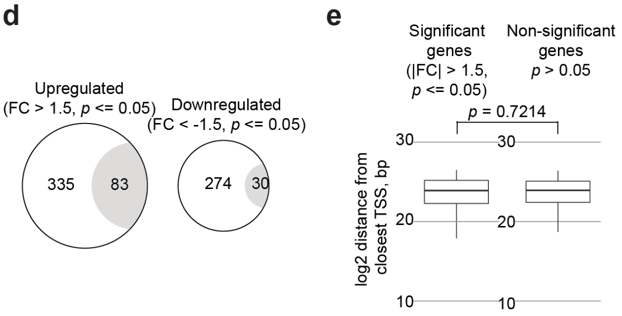

f

MPP8 removal (2i/LIF, 6 h IAA)			MPP8 removal (2i/LIF, 48 h IAA)		
LINE class	Fold change	p_{adj}	LINE class	Fold change	p_{adj}
L1Md_Gf	6.85	1.35E-25	Lx2A	5.25	3.26E-06
L1_Md_T	3.09	3.88E-17	L1Md_T	4.67	1.85E-08
L1Md_F	2.46	3.32E-25	L1Md_A	2.50	6.25E-03
L1Md_A	2.18	7.96E-11	L1_Mm	2.35	4.10E-03
L1_Mur3	1.84	1.51E-03			
Lx9	1.80	4.68E-04			
Lx8b	1.70	4.77E-02			
L1_Mus3	1.49	1.54E-02			
Lx8	1.46	4.31E-02			

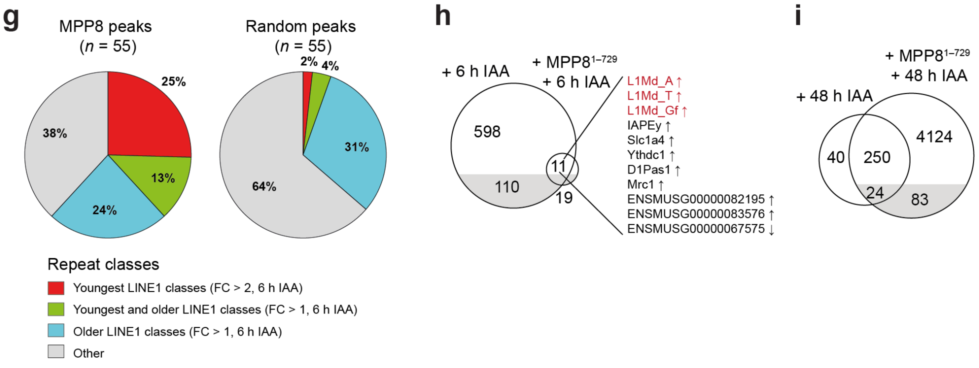

Supplementary Figure 7. De-repression of LINE1 elements correlates with the loss of self-renewal in ground-state mESCs. a Fractions of the top 10 enriched LINE classes (LINE1 class count /total LINE1 count) overlapping MPP8 peaks (n = 55, mean length = 11097 bp), as well as their fractions within all annotated LINE1 classes present in mm10 and overlapping random sampled peaks (n = 55, mean length = 11097 bp). **b** qRT-PCR analysis of *Mphosph8*, evolutionary young L1A class LINE1 elements, *L1Orf2* encoding LINE1-encoded ORF2 protein and SINE transcript expression in Cas9-expressing mESCs transduced with and constantly selected for sgRNA *Mphosph8* 24 or NegControl for a time frame equivalent to competition assay time-point day 6 (NegControl: n = 2, *Mphosph8* sgRNA 24: n = 3 biologically independent samples). **c** Same as in (**b**) but in wildtype (Wt) MEFs or two *Mphosph8* knockout clones 3 (KO3) and 8 (KO8) (mean ± s.d., n = 3 biologically independent samples). p = 0.0001 (KO3) and p = 0.0002 (KO8) for Mphosph8, p = 0.1596 (KO3) and p = 0.7101 (KO8) for L1A, p = 0.0347 (KO3) and p = 0.9944 (KO8) for L1orf2, p = 0.1593 (KO3) and p = 0.8596 (KO8) for SINE (two-tailed unpaired Student's t-test comparing wt to KO3 or KO8). **d** Number of transcripts up- or downregulated upon 6-hour auxin treatment in $Mpp8^{mAID}$; OsTIR1 cells ($|FC| > 1.5$, $p < 0.05$). Grey area indicates transcripts that are not rescued by $MPP8^{wt}$ expression. **e** Log2 distance between high-confidence MPP8-bound regions (n = 55) and closest TSS of significantly differentially expressed ($|FC| > 1.5$, $p < 0.05$) genes (n = 698) or non-significant ($p > 0.05$) genes (n = 21808) upon 6-hour auxin treatment of $Mpp8^{mAID}$; OsTIR1 cells. The boxes designate the 25th and 75th percentiles and are divided be the median; the whiskers extend to the 5th and 95th percentiles. p = 0.7214 (two-sided Wilcoxon test). **f** Summary of significantly upregulated LINE1 classes (FC > 1, $p < 0.05$) after 6-hour or 48-hour auxin treatment in $Mpp8^{mAID}$; OsTIR1 cells grown in 2i/LIF. **g** Overlap of 55 high-confidence MPP8 peaks (n = 55, mean length = 11097 bp) or random sampled peaks (n = 55, mean length = 11097 bp) with LINE1 classes transcriptionally upregulated after 6-hour auxin treatment. L1Md_Gf, L1Md_A and L1Md_T classes were considered youngest LINE1 classes (FC > 2, $p < 0.05$) and the remaining upregulated LINE1 classes (FC > 1, $p < 0.05$) were classified as older LINE1 classes. Several peaks overlapped more than one annotated repeat element. **h, i** Overlap of transcripts

differentially expressed after 6-hour (**h**) or 48-hour (**i**) auxin treatment in Mpp8^{mAID}; OsTIR1 cells and cells additionally expressing MPP8$^{1–729}$. Transcripts that overlap and are rescued by expression of wildtype MPP8 are listed and LINE1 elements highlighted in red. Grey area indicates transcripts that are not rescued by MPP8^{wt} expression. Overlapping LINE1 classes are highlighted in red. *$p < 0.05$, **$p < 0.01$, ***$p < 0.001$, ****$p < 0.0001$, ns = not significant. FC = fold change. Source data are provided as a Source Data file.

Supplementary Fig. 8

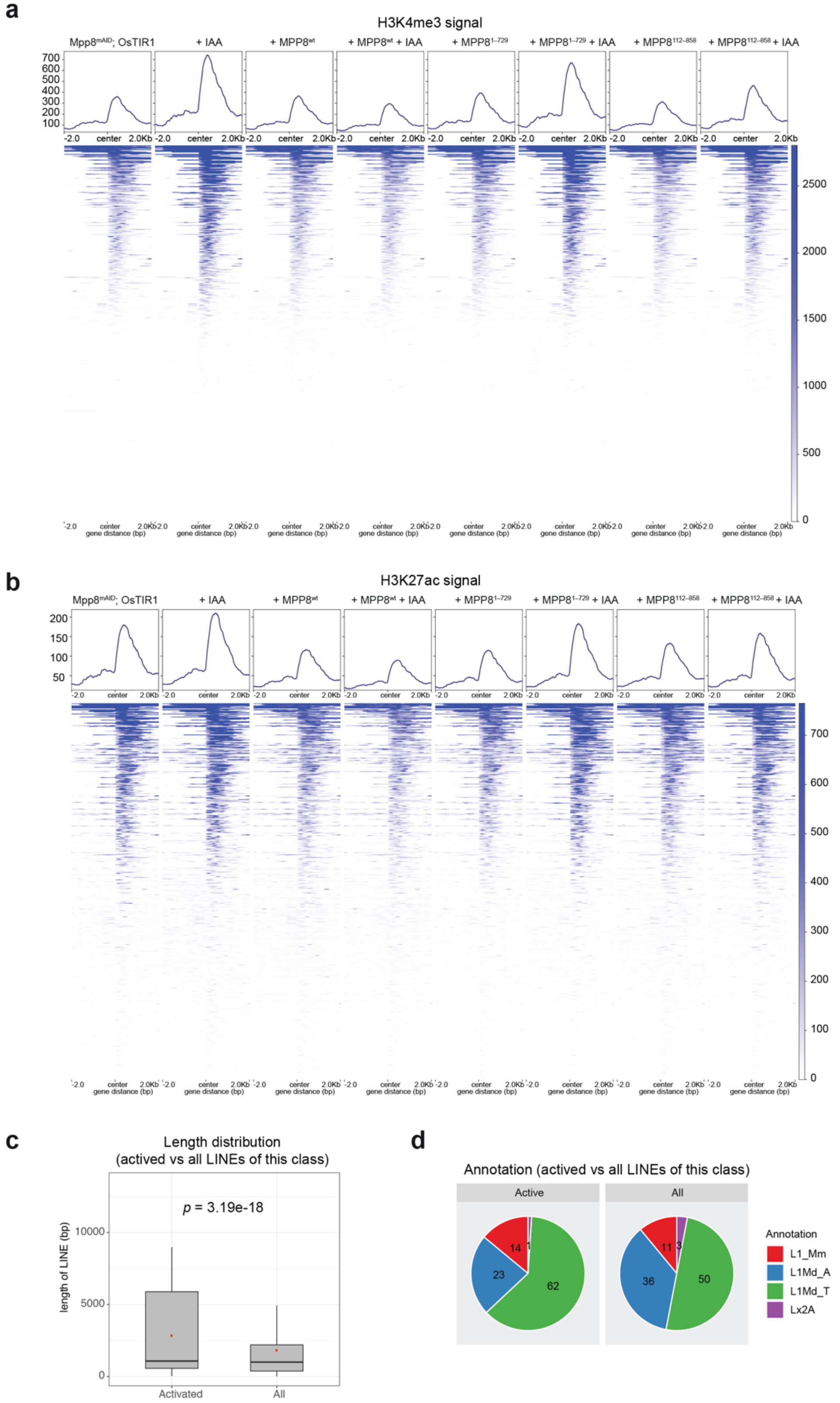

Supplementary Figure 8. ChIP-seq analysis of H3K4me3 and H3K27ac on MPP8-repressed LINE1 classes. a Composite profiles (top) and density plots (bottom) showing enrichment of H3K4me3 at the TSS center ± 2 kb of activated LINE1 elements. Activated LINE1 elements were defined as belonging to transcriptionally upregulated (FC > 1.5, $p < 0.05$) classes and showing an increase of at least two-fold in H3K4me3 at 48 hours after auxin-mediated MPP8 depletion ($n = 512$). **b** Composite profiles (top) and density plots (bottom) showing enrichment of H3K27ac at the transcription start site (TSS) center ± 2 kb of activated LINE1 elements. **c** Box plots showing length distribution of activated LINE1 elements and all LINEs part of transcriptionally upregulated (FC > 1.5, $p < 0.05$) classes at 48 hours after auxin-mediated MPP8 depletion (center bar = median, red dot = mean). The lower and upper hinges correspond to the first and third quartiles, and the whiskers extend to the most extreme values within the bounds of 1.5 * the interquartile range. $n = 2$ biologically independent replicates for RNA-seq and $n = 1$ sample for ChIP-seq per condition. $p = 3.18e-18$ (one-sided Mann-Whitney U test). **d** Pie charts showing the annotation of activated LINE1 elements and all LINEs part of transcriptionally upregulated (FC > 1.5, $p < 0.05$) classes at 48 hours after auxin-mediated MPP8 depletion.

Supplementary Fig. 9

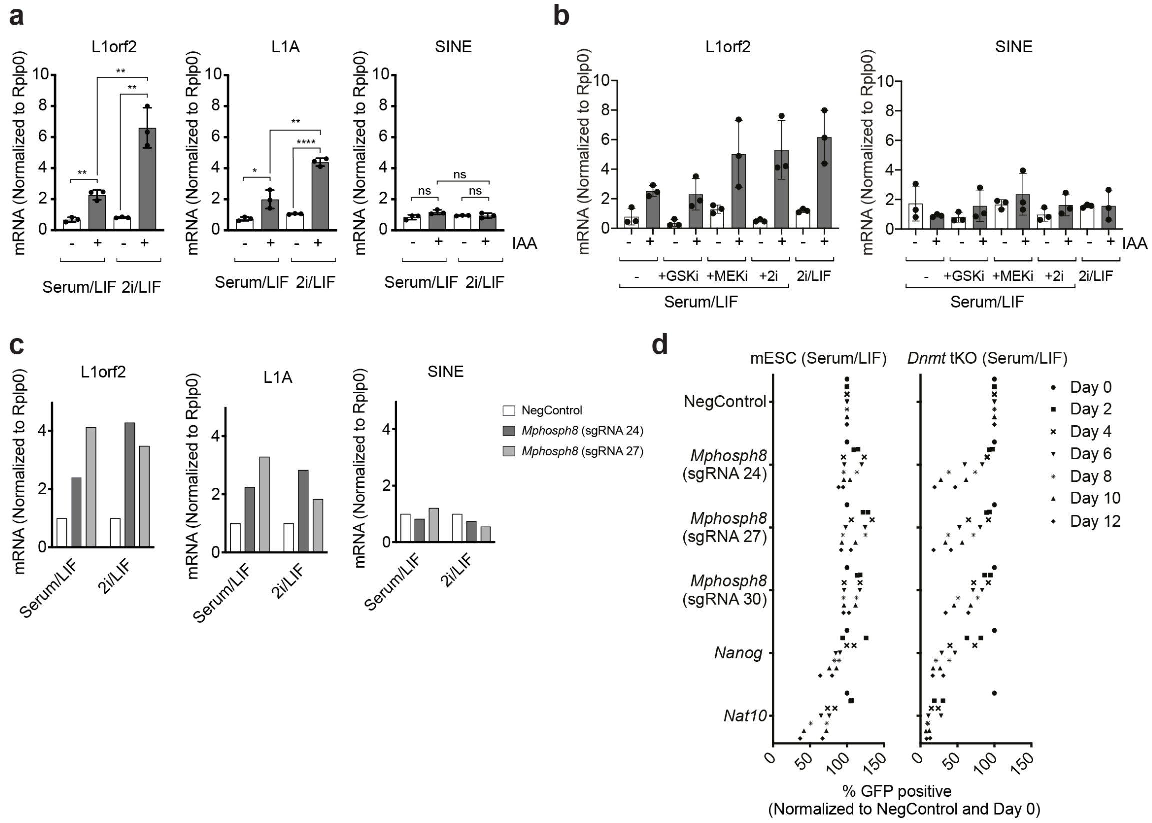

Supplementary Figure 9. DNA methylation protects serum/LIF-grown mESCs from cell death upon MPP8 removal. a qRT-PCR analysis of L1A elements, L1ORF2-encoding transcript and SINE transcript expression in $Mpp8^{mAID}$; OsTIR1 or $Mpp8^{mAID}$ (control) cells cultured under either 2i/LIF or serum/LIF culture conditions ± 500 μM IAA treatment (mean ± s.d., n = 3 biologically independent samples). p = 0.0014 (Serum/LIF ± IAA), p = 0.0015 (2i/LIF ± IAA) and p = 0.0049 (Serum/LIF vs 2i/LIF) for L1orf2, p = 0.0230 (Serum/LIF ± IAA), p = 2.40e-05 (2i/LIF ± IAA) and p = 0.0031 (Serum/LIF vs 2i/LIF) for L1A, p = 0.0547 (Serum/LIF ± IAA), p = 0.9939 (2i/LIF ± IAA) and p = 0.1607 (Serum/LIF vs 2i/LIF) for SINE (two-tailed unpaired Student's *t*-test,). **b** qRT-PCR analysis of L1ORF2-encoded transcript and SINE transcript expression in $Mpp8^{mAID}$; OsTIR1 cells cultured in serum/LIF, serum/LIF/GSKi, serum/LIF/MEKi, serum/LIF/2i or 2i/LIF culture conditions ± 500 μM IAA treatment (mean ± s.d., n = 3 biologically independent samples). **c** qRT-PCR analysis of L1A elements, L1ORF2-encoding transcript and SINE transcript expression in Cas9-expressing *Dnmt* tKO

(knockout for *Dnmt3a*, *Dnmt3a* and *Dnmt1*) mESCs transduced with sgRNA 24, 27 or 30, targeting *Mphosph8* or sgRNAs targeting *Nanog* and *Nat10* as positive control. A non-targeting sgRNA (NegControl) served as negative control. Cells were sorted for GFP at a time-point equivalent to competition assay day 6 (n = 1). **d** Competition-based proliferation assays in indicated Cas9-expressing cell lines grown in serum/LIF. sgRNAs targeting the core essential gene *Nat10* and mESC-essential gene *Nanog* served as positive controls while a non-targeting sgRNA (NegControl) served as negative control. The percentage of GFP+ cells is normalized to the day zero measurement and the measurement of NegControl at the respective day (n = 2 independent experiments). *p < 0.05, **p < 0.01, ***p < 0.001, ****p < 0.0001, ns = not significant. Source data are provided as a Source Data file.

Supplementary Fig. 10

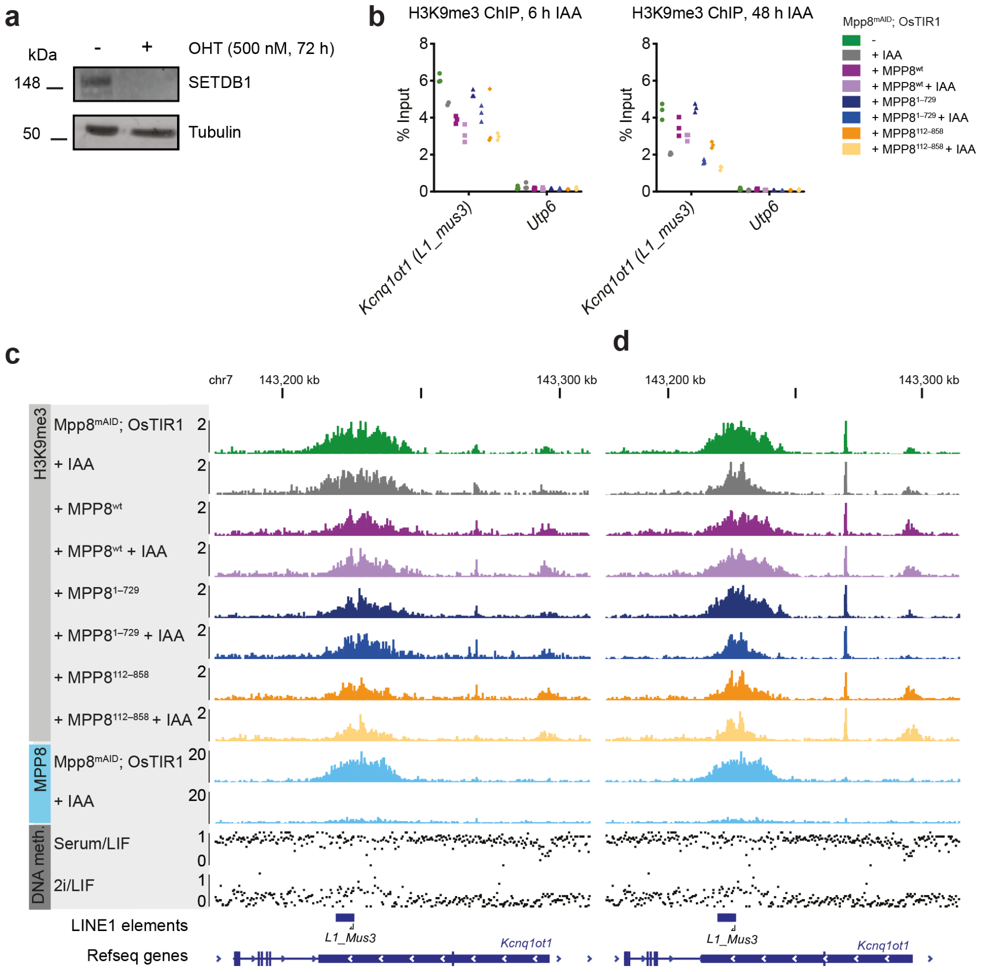

Supplementary Figure 10. Changes of H3K9me3 at MPP8-binding sites of wildtype and mutant versions of MPP8-expressing cells. a Western blot analysis of SETDB1 or ßACTIN (loading control) in conditional *Setdb1* knockout cells treated with 500 nM 4-hydroxytamoxifen (OHT) for 72 hours and untreated control (*n* = 1). **b** ChIP-qPCR validation of ChIP-seq data shown in Supplementary Fig. 9c and d, respectively, at a known MPP8 target locus (*L1_mus3*, *Kcnq1ot1*) and non-target locus (*Utp6*) (*n* = 2 or 3 technical replicates). **c, d** Representative genome browser tracks of: ChIP-seq signal generated with a H3K9me3-specific antibody. Enrichment is shown for Mpp8^{mAID}; OsTIR1 cells additionally expressing MPP8^{wt}, MPP8$^{1–729}$, MPP8$^{112–858}$, respectively, ± 500 µM IAA for 6 hours (**c**) or 48 hours (**d**); ChIP-seq signal generated with an MPP8 antibody in Mpp8^{mAID}; OsTIR1 cells ± 500 µM IAA for 16 hours; DNA methylation

profiles in mESCs grown in serum/LIF or 2i/LIF culture conditions (0 = unmethylated, 1 = fully methylated CpG sites; data taken from Habibi et al., 2013). Repeatmasker track showing the location of relevant LINE1 elements is indicated at the bottom. Source data are provided as a Source Data file.

Supplementary Fig. 11

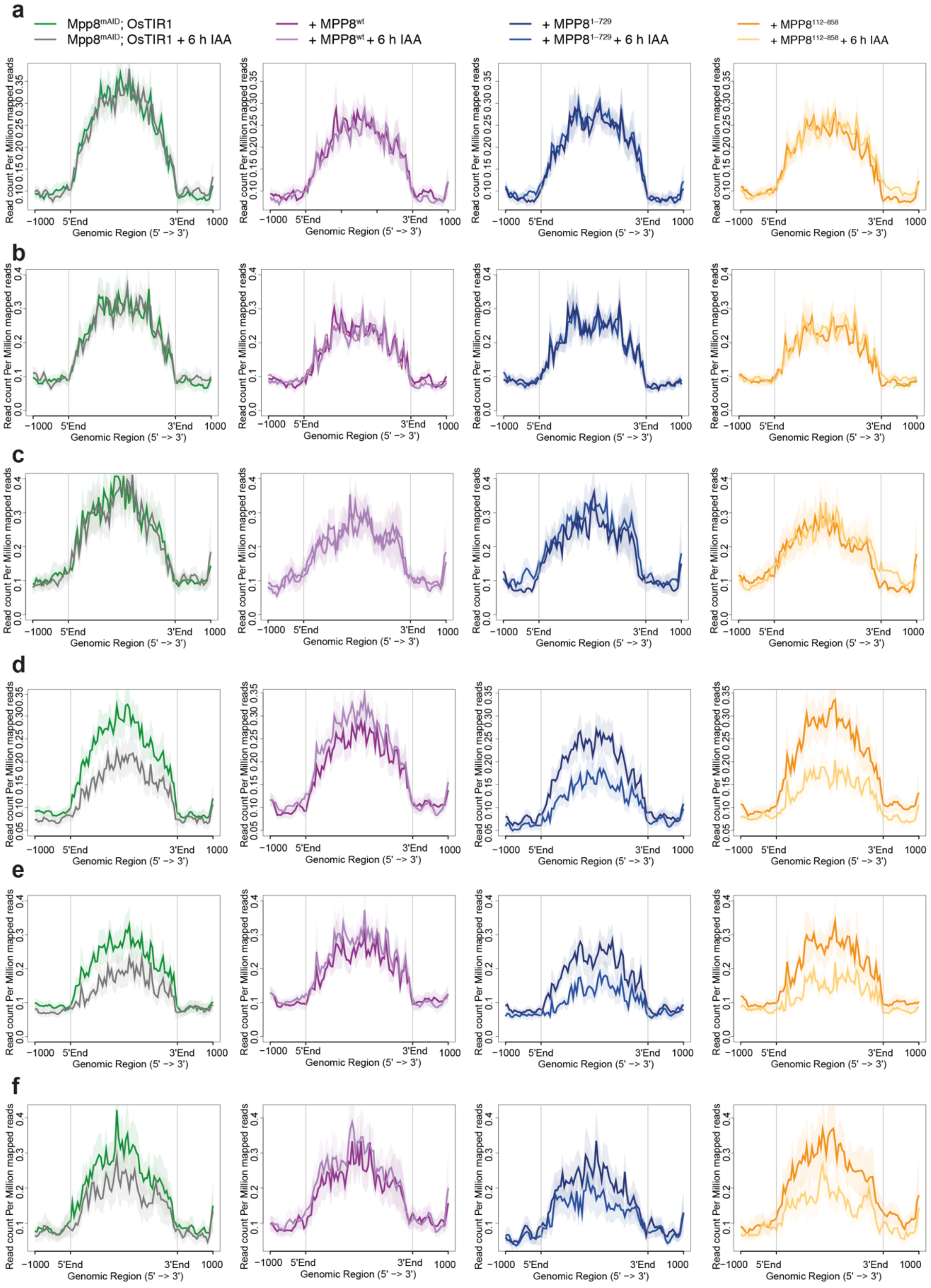

Supplementary Figure 11. H3K9me3 average profiles over MPP8-binding sites of wildtype and mutant versions of MPP8-expressing cells. a-c Aggregate plot comparing the average H3K9me3 ChIP-seq signal after 6 hours of MPP8 induced degradation, corresponding to data shown in Fig. 6c: Average H3K9me3 ChIP-seq signal over all identified MPP8 peaks (n = 55) (**a**), MPP8 peaks overlapping LINE1 elements significantly upregulated after 6 hours of MPP8 induced degradation (n = 34) (**b**) and the remaining MPP8 peaks non-overlapping upregulated LINE1 elements (n = 19) (**c**). **d-e** Aggregate plot comparing the average H3K9me3 ChIP-seq signal 48 hours after MPP8 induced degradation, corresponding to data shown in Fig. 6d: Average H3K9me3 ChIP-seq signal over all identified MPP8 peaks (n = 55) (**d**), MPP8 peaks overlapping LINE1 elements significantly upregulated after 6 hours of MPP8 removal (n = 34) (**e**) and the remaining MPP8 peaks non-overlapping upregulated LINE1 elements (n = 19) (**f**).

Supplementary Fig. 12

a

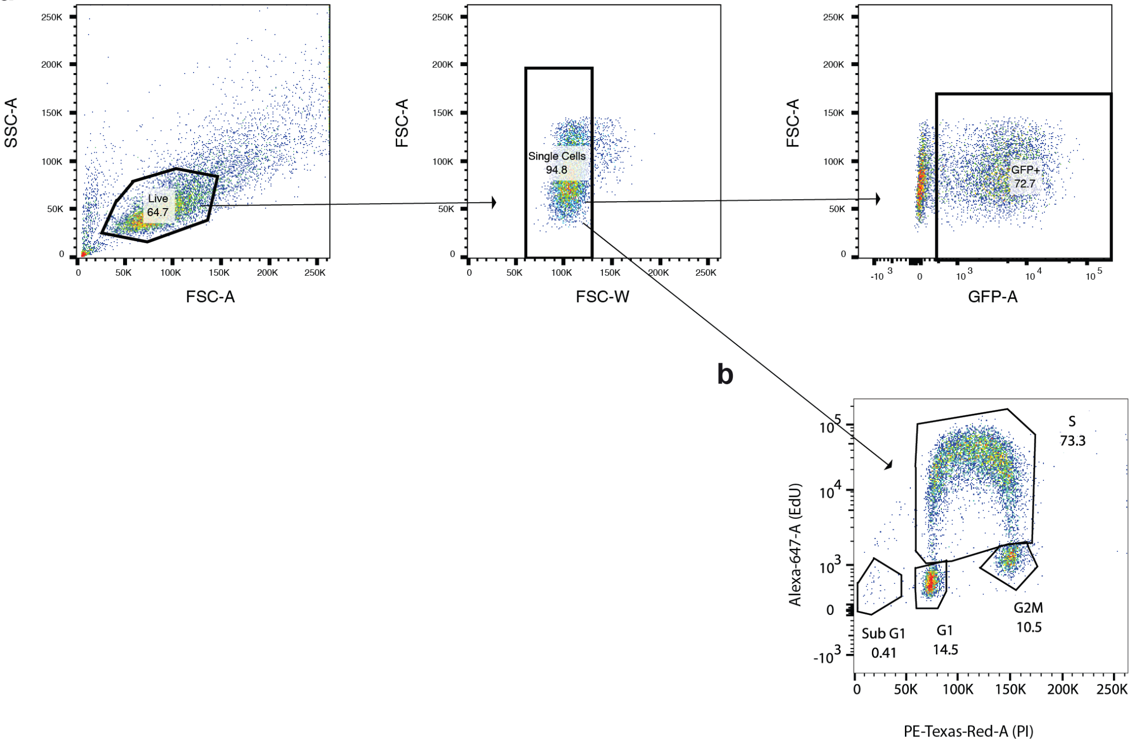

Supplementary Figure 12. Example FACS gating strategy used for competition-based proliferation assays and cell-cycle analysis. a Live cells were selected (forward scatter area (FSC-A) versus side scatter area (SSC-A)), followed by selection of single cells (forward scatter width (FSC-W) versus height (FSC-H)) and the percentage of GFP+ cells was assessed. Non-transduced cells were used as gating controls. Data presented in Fig. 1e-g; 3c; 4e and Supplementary Fig. 1f; 2a, b; 9d correspond to the value assessed as GFP+-gated cells. **b** Four cell cycle populations, SubG1, G1, S and G2M, were assessed via gating of PE-Texas-Red (PI) versus Alexa-647 (EdU). Data presented in Fig. 2e correspond to the values assessed as SubG1-, G1-, S- and G2M populations, respectively.

Supplementary Table 1. Hits previously identified as mESC identity factors

Hit identified	Essential role in mESCs	Source
Ep400<br>Kat5	Tip60-p400 complex<br>Histone acetylation	Fazzio et al., 2008
L3mbtl2	Ring2-L3MB5LC complex	Qin et al., 2012
Thap11 (Ronin)	Metabolism	Dejosez et al., 2009
Trim28	Myc/Zfx cofactor	Fazzio et al., 2008<br>Hu et al., 2009
Setdb1	Silencing of lineage-specific regulators	Bilodeau et al., 2009
Lin9	DREAM complex	Esterlechner et al., 2013
Ncl		Ding et al., 2009<br>Yang et al., 2011
Nfyb		PNAS 2013 Cinghua
Nipbl	Cohesin; Core circuitry	Fazzio et al., 2008
Ogt		Jang et al., 2012
Rbbp6		Li et al., 2007
Rbx1		Jia and Sun, 2009
Ppp4c		Hu et al., 2009

Supplementary Table 2. Oligos used for knockin of endogenous miniAID tag on 3'UTR of *Mphosph8* genomic locus.

Oligo name	Sequence
gBlocks	
**Mpp8_mAID_Left_Block**	TAAAACGACGGCCAGTGAATTCGATGTATATTAGTGAACTTCAGACAATCCCCACTCACTATAAAAGAAAATGATCCAAATTGTTAGAACATGTTAATGTTTATTCACTTAAATCGTGCTACCATATGTCCTACAGAAAACAATGTGAGACTAGAATAGAATAGTAGCATGCCACGCCCTCATTCTAACACACTCTGCTGCTGGAACTCTTCCAGAGGCTAATAAAATGAACACCTTGTATTGAATGCAGTCATGTGTTGTTTTTATTTCAGCCCTAGGTAGCCTAGAACTCTTTAGACATGGTAAGCCTTGAAGGTGAGGTTTTTGGGGGGCTGCACAGAGAGATCCTATCTTAAAATTCAAAAACAAAACCCCTCAGTTCGCTGATCGGAGTTCCTGGTCTCTATTGTAAATGTCACAGCCCTGACTTGAGTCTGACTTTATCTTCTCTCTTTCCCCTCTAGGACAGTCACTTTGTTTATTCATTCAGTCCTGTTGCTGGCCCCAATAAGTTGTTTATAAGGTTGACGGAAGCACCCTTTGCCAAGGTGAAAATCTTTGCTTCATATTTACACAGTAACTGCTGACATAATAATAGCAATACTAATAAATCCTCTCACTAGAAACGTCAGGAGACATGGTTCAGACAAGCTCTTTGTACTAGACGGCTTATGTTGGGCAAACTTCGGCTCTCTGAGGCAGCTATGCATAGCATATTTGGCAAGTCTGGGGGCATCCCTGCTGAGCTCTCCAAATCTTGACTTCAGTACTAAAATGCCACTACCCTCACCTAGGACAGATCCCTCCATATGAAAAAAGGAAGCTGGACTACTTAGAAGACCATTCTAATAATGGTTACCTAATATATATTATAGCAATAACTTGGATGTTCTTACAAGGTGGAATTTGTGTACTGACATTCATTCTCTTCTTTAGGTTAAGTTGCTAATAGGTGCATACAGAGTACAGCTGCAGGGTGGTTCTGGTAAGGAGAAGAGTG
**mAID P2A BFP middle**	GGTGGTTCTGGTAAGGAGAAGAGTGCTTGTCCTAAAGATCCAGCCAAACCTCCGGCCAAGGCACAAGTTGTGGGATGGCCACCGGTGAGATCATACCGGAAGAACGTGATGGTTTCCTGCCAAAAATCAAGCGGTGGCCCGGAGGCGGCGGCGTTCGTGAAGGTATCAATGGACGGAGCACCGTACTTGAGGAAAATCGATTTGAGGATGTATAAAGGATCCGGCGCAACAAACTTCTCTCTGCTGAAACAAGCCGGAGATGTCGAAGAGAATCCTGGACCGGTGTCTAAGGGCGAAGAGCTGATTAAGGAGAACATGCACATGAAGCTGTACATGGAGGGCACCGTGGACAACCATCACTTCAAGTGCACATCCGAGGGCGAAGGCAAGCCCTACGAGGGCACCCAGACCATGAGAATCAAGGTGGTCGAGGGCGGCCCTCTCCCCTTCGCCTTCGACATCCTGGCTACTAGCTTCCTCTACGGCAGCAAGACCTTCATCAACCACACCCAGGGCATCCCCGACTTCTTCAAGCAGTCCTTCCCTGAGGGCTTCACATGGGAGAGAGTCACCACATACGAAGACGGGGGCGTGCTGACCGCTACCCAGGACACCAGCCTCCAGGACGGCTGCCTCATCTACAACGTCAAGATCAGAGGGGTGAACTTCACATCCAACGGCCCTGTGATGCAGAAGAAAACACTCGGCTGGGAGGCCTTCACCGAGACGCTGTACCCCGCTGACGGCGGCCTGGAAGGCAGAAACGACATGGCCCTGAAGCTCGTGGGCGGGAGCCATCTGATCGCAAACGCCAAGACCACATATAGATCCAAGAAACCCGCTAAGAACCTCAAGATGCCTGGCGTCTACTATGTGGACTACAGACTGGAAAGAATCAAGGAGGCCAACAACGAGACCTACGTCGAGCAGCACGAGGTGGCAGTGGCCAGATACTGCGACCTCCCTAGCAAACTGGGGCACAAGCTTAATTAA
**Mpp8_mAID_Right_Block**	CAAACTGGGGCACAAGCTTAATTAACCACAAAGGATGCAGAACCGGCCTCTTTTCAGCTGGATTCCAAGGACCATCCCTGTAGCAGTTTGGAATTCTGGTGCAGCTCAAATGTTCCTACCTAGTTGTAAACTTAAAATTTAAGCCTGCTGCCTGTTCAGGTTTGTCTACATATATTTGTGCCATTGGTCGAAAACGTTCAATGTTCTGCTTAGTTTATTTCCACAATGTGGAACAGTACGTGTAAGAAATATTTAAGGAAATTAAAAACCTTTTGAATTGAAATTTTCTACTAGTCGTTGTGAGTTTGTATTTGAAAAACAAAAACCAGATTCAGGGTGGGCCCCACTACTGAGTTCAAGAGTAGCCATGTCTACAACAACATGAACCTTTTACAAATCAGGTTTAAACATTTACTATACAACTAAGTTTGATATTCTTAAAGGTGGAAAAGCAACTTAATTATAATTATAAAATGTAACAATGCAGCTGCAGGATTTTCCTCTGAGCCTCTTTGTTTCCAGAATAAAGACATCTGAGATTGAATTATAAGCTTGGGCTTGTTCTGACTCGCTTGTAACTTAACCAACACGTTTATTCTATTCCATGGTCAGAGGCAATTCTGTCCCCTCACCCGATTATCCCAGAGTCCTCTTCCCTACCAGAACTTCCACCTATTTCCTTCCTGCCTCAGCTAGCAGGCCATCAGCTACTTATTTATGGCAGGTGATGCTTCCATACAGTACACAACAGATTCTCTCTACAGTAGAAATGTGTCCCGTAGCATACTCTTGACATGTGCTGTGACTGCTCATAAGCATAAATCACAAAATAAGGTTTTCTGCCTGCGGCTTTTGCGTCATTAGGAGTTTTTCCTCACAGCGTGCTGTAACCTGTAAGAGACAAAGCTTTCATCAATAAATGGGAATGGTATACATGTGCACACATGCATGCATTTTGTCCTTTGTAACACAGCCATCAAGCTTGGCGTAATCATGGTCA
Sequencing primers for verification of cloning	
**pUC19_R**	GTTAGCTCACTCATTAGGCAC
**mAID_BFP_R**	TTAATTAAGCTTGTGCCCC
**mAID_BFP_F**	GTGAACTTCACATCCAACGG
**mAID_R**	ATTGATACCTTCACGAACGC
**mAID_F**	AAGGAGAAGAGTGCTTGTCC
**pUC19_F**	AACTGTTGGGAAGGGCGATC
Screening of successfully targeted clones	
**Outside LeftHA/Outside RightHA**	
**Mpp8_outside_Left_HA_F1**	*GTGAATGCTGGGCATACGTG*
**Mpp8_Outside_Right_HA_R1**	*ACACAGTAACTGAGGCGTGG*
**Left junction**	

**Mpp8_outside_Left_HA_F1**	*GTGAATGCTGGGCATACGTG*
**Mpp8_mAID_Inside_R**	*AGGAAACCATCACGTTCTTCCG*
**Right junction**	
**Mpp8_mAID_Inside_F**	*ATGCCTGGCGTCTACTATGTGG*
**Mpp8_Outside_Right_HA_R1**	*ACACAGTAACTGAGGCGTGG*
**Outside mAID Left/Outside mAID Right**	
**Mpp8_qPCR_Left_F**	*CCCTGCTGAGCTCTCCAAAT*
**Mpp8 Flag2 3'UTR_R**	GGAACATTTGAGCTGCACCAG
**Oligos used for cloning of sgRNA targeting 3'UTR**	
**Mpp8 3'sgRNA_2_woG_F**	CACCGCTGCAGTGACCACAAAGGA
**Mpp8 3'sgRNA_2_woG_R**	AAACTCCTTTGTGGTCACTGCAGC

Supplementary Table 3. Oligos for knockin of endogenous Flag2 tag on 3'UTR of *Mphosph8*, *Pphln1* and *Tasor* genomic loci, respectively

Oligo name	Sequence
ssODN knockin	
**Mphosph8**	gtgtactgacattcattctcttctttagGTTAAGTTGCTAATAGGTGCATACAGAGTACAGCTGCAGGGCGCCGACTACAAG<br>GACGACGATGACAAGGACTACAAGGACGACGATGACAAGCGTACCGGTTGACCACAAAGGATGCAGAACC<br>GGCCTCTTTTCAGCTGGATTCCAAGGACCATCCCTGTAGCAGTTTGGA
**Pphln1**	CGTGGAAGAGCTCAAGCGTTTCATTACTGAGTATGACAACTCTGCTCAGGACTTTGGAGACCCCTTTGGCG<br>CCGACTACAAGGACGACGATGACAAGGACTACAAGGACGACGATGACAAGCGTACCGGTTAGAAGAAGAA<br>GTACCCACGTCAAAAGGTTTTCTTAGATTGTTTTTTTCTCCTTGGATTGTGTAAATCCT
**Tasor**	ttaccgtgtaactttagatgctgttttgacattaaccccttggagctgggagttgggatttcGcaacatGGCGCCGACTACAAGGACGACGATG<br>ACAAGGACTACAAGGACGACGATGACAAGCGTACCGGTtaaacgtgaacacatttcgcagaagcttttagcatcggattatctggac<br>actcacacctagtgtgag
Oligos for sgRNA cloning into PX458	
**Mphosph8 3' sgRNA_2 F**	CACCGGCTGCAGTGACCACAAAGGA
**Mphosph8 3' sgRNA_2 R**	AAACTCCTTTGTGGTCACTGCAGCC
**Pphln1_PX458_1_F**	CACCGTTTTAGAAGAAGAAGTACCC
**Pphln1_PX458_1_R**	AAACGGGTACTTCTTCTTCTAAAAC
**Tasor_PX458_1_F**	CACCGAATGTGTTCACGTTTAATGT
**Tasor_PX458_1_R**	AAACACATTAAACGTGAACACATTC
Screening primers for successful knockin	
**Mpp8Flag2_endtag_intron_F1**	AAAGGAAGCTGGACTACTTAGAAGA
**Mpp8Flag2_endtag_Flag2_R1**	CGTCCTTGTAGTCCTTGTCATCG
**Mpp8Flag2_endtag_Flag2_F1**	GCGTACCGGTTGACCACA
**Mpp8Flag2_endtag_3UTR_R1**	GGAACATTTGAGCTGCACCAG
**Pphln1 end tag intron F**	AGACTGCGAGACTTTCGGGA
**Pphln1 end tag flag2 R**	GTCCTTGTAGTCCTTGTCATCGTC
**F Pphln1endog tag flag2 F**	ACGACGATGACAAGCGTACC
**Pphln1endog tag 3'UTR R**	ATTCTACACATAGGGGAAACATTCT
**F Intron Tasor endog tag F**	tcagtgcagctacatgtcagaa
**Tasor flag2 endog R**	CGTCCTTGTAGTCCTTGTCATCG
**F Tasor end tag Flag2 F**	CGACGATGACAAGGACTACAAGGA
**Tasor end tag R 3'UTR**	CTTAAAGCTTGGTCTTCTGCCAC

Supplementary Table 4. Primers used for sgRNA vector cloning, library amplification and for adapter sequences used in high-throughput sequencing of the CRISPR/Cas9 library

Oligo name	Sequence
**<u>Cloning of the U6-sgRNA-SFFV-puro-P2A-EGFP vector for the Epi screen</u>**	
**BamHI-Kozak-PuroR-Nhel_1 F**	TGAGTCGGATCCGCCACCATGACCGAG
**BamHI-Kozak-PuroR-Nhel_1 R**	GCTGCCGCTAGCGGCACCGGGCTTG
**<u>Array oligo library amplification</u>**	
**CRISPR ArrayF**	TTACTATACGTCTCCCACCG
**CRISPR ArrayR**	CTGGACTCACGTCTCAAAAC
**<u>Addition of Illumina adapter sequences for high-throughput sequencing</u>**	
**Epi_Library_Illumina_F**	AATGATACGGCGACCACCGAGATCTACACTCTTTCCCTACACGACGCTCTTCCGATCTTTAGGCTCTTGTGGAAAGGACGAAACACC
**Epi_Library_Illumina_R702**	CAAGCAGAAGACGGCATACGAGATCGATGTGTGACTGGAGTTCAGACGTGTGCTCTTCCGATCTATTCTTTCCCCTGCACTGTACC
**Epi_Library_Illumina_R703**	CAAGCAGAAGACGGCATACGAGATTTAGGCGTGACTGGAGTTCAGACGTGTGCTCTTCCGATCTATTCTTTCCCCTGCACTGTACC
**Epi_Library_Illumina_R706**	CAAGCAGAAGACGGCATACGAGATGCCAATGTGACTGGAGTTCAGACGTGTGCTCTTCCGATCTATTCTTTCCCCTGCACTGTACC
**Epi_Library_Illumina_R707**	CAAGCAGAAGACGGCATACGAGATACTTGAGTGACTGGAGTTCAGACGTGTGCTCTTCCGATCTATTCTTTCCCCTGCACTGTACC
**Epi_Library_Illumina_R709**	CAAGCAGAAGACGGCATACGAGATGATCAGGTGACTGGAGTTCAGACGTGTGCTCTTCCGATCTATTCTTTCCCCTGCACTGTACC
**Epi_Library_Illumina_R710**	CAAGCAGAAGACGGCATACGAGATTAGCTTGTGACTGGAGTTCAGACGTGTGCTCTTCCGATCTATTCTTTCCCCTGCACTGTACC
**Epi_Library_Illumina_R711**	CAAGCAGAAGACGGCATACGAGATGGCTACGTGACTGGAGTTCAGACGTGTGCTCTTCCGATCTATTCTTTCCCCTGCACTGTACC

Supplementary Table 5. Oligos used for sgRNA cloning of sgRNAs not included in the library

Oligo name	Sequence
**Pou5f1_sgRNA_POU F**	CACCGGGCCTCGAAGCGACAGATGG
**Pou5f1_sgRNA_POU R**	AAACCCATCTGTCGCTTCGAGGCCC
**Pou5f1_sgRNA_Homeo F**	CACCGACTCACATCGCCAATCAGCT
**Pou5f1_sgRNA_Homeo R**	AAACAGCTGATTGGCGATGTGAGTC
**Pphln1_sgRNA_2 F**	CACCGAGACTGCGAGACTTTCGGGA
**Pphln1_sgRNA_2 R**	AAACTCCCGAAAGTCTCGCAGTCTC
**Tasor_sgRNA_2 F**	CACCGATAGGCTCTATATGCCAAGA
**Tasor_sgRNA_2 R**	AAACTCTTGGCATATAGAGCCTATC
**Morc2a_sgRNA_1 F**	CACCGCCACCTGGAATGCTCGAACA
**Morc2a_sgRNA_1 R**	AAACTGTTCGAGCATTCCAGGTGGC
**Atf7ip_sgRNA_3 F**	CACCGAGAATGGCATTGTTCTGTCG
**Atf7ip_sgRNA_3 R**	AAACCGACAGAACAATGCCATTCTC

Supplementary Table 6. Primers for assessing sgRNA targeting efficiency by TIDE

Oligo name	Sequence
**Nat10 genomic F**	ACACGTTAAATCACCCCCTTGA
**Nat10 genomic R**	TGTATCCAGCCCAAACCCTTT
**Naa20 genomic F**	GCACCCGAATCCAGTGTAAATCA
**Naa20 genomic R**	ACAAGGAATGATCCACAAAGACTAC
**Naa50 genomic F**	AGACAGTGTAAATGTTGAGGCCAT
**Naa50 genomic R**	GCAAATTCCTACCTGACTTCCCAA
**N6amt1_26 genomic F**	TGTACGGGCACGTGGGTC
**N6amt1_26 genomic R**	TGGTTGCTAAACTCCGGCTG
**N6amt1_51 genomic F**	CCCAACACCATGTTCGAGTT
**N6amt1_51 genomic R**	GTATTTCCACTAAAAGTGACTGACC
**Hlcs_125 genomic F**	CCTACCAAAGGCTGTGAGGG
**Hlcs_125 genomic R**	GGAGACTGCCTGTGCTCTAC
**Hlcs_103 genomic F**	TCTCATGCCCTGTGGTCGAT
**Hlcs_103 genomic R**	CATCCGGTCCCCTCTGTATCA
**Wbscr22_ genomic 37 F**	ACCCCTTTTGTGAGGAGCTG
**Wbscr22_ genomic 37 R**	ACAGCCAGGTTTAAGAAACGC
**Wbscr22_ genomic 17 F**	GTAGAAAGAGTCCCTCTAACTGATT
**Wbscr22_ genomic 17 R**	GATGTCAATGCCCACCCAGTA
**Mphosph8 30 genomic F_2**	GTGCCTTGCCAGTTTTTCGT
**Mphosph8 30 genomic R_2**	CTGGTGCTTCCCGTCAGAG
**Mphosph8 _24 genomic F_1**	CAACCTAACCTAAGTGGATCCTT
**Mphosph8 _24 genomic R_1**	GCAGTGTAGCTAGAAGTTGTGC
**Mphosph8 _24 genomic F_2**	CAGTGAACCTATTTCTGGCCTCTA
**Mphosph8 _24 genomic R_2**	GCCGATTAGGCAAGTCACACT
**Mphosph8 _27 genomic F_1**	CTGTCTGTTGCCAGTGCTTG
**Mphosph8 _27 genomic R_1**	ATGGTACGTTCGTTCGCCTG

Supplementary Table 7. Primers used for cloning of ectopic Mpp8 targeting vectors

Oligo name	Sequence
Addition of N-terminal Kozak and C-terminal double-flag tag	
PCR1	
**Kozak Mphosph8 F**	GCC ACC ATG GCG GCG GCG
**Mphosph8 Flag2 Part1 R1**	ATCGTCGTCCTTGTAGTCGGCGCCCTGCAGCTGTACTCTGTATGCACC
PCR2	
**Kozak Mphosph8 F**	GCC ACC ATG GCG GCG GCG
**Flag2 Part2 R**	CTAACCGGTACGCTTGTCATCGTCGTCCTTGTAGTCCTTGTCATCGTCGTCCTTGTAG TCGGCG
Cloning of ectopic Mpp8(mutant)flag2 vectors	
**Mphosph8 *M1-E729 Flag2***	
**C-terminal_deletion_F**	GGCGCCGACTACAAGGAC
**M1_E729_R**	TTCTGCAACTCTGGAAAGTGTC
**Mphosph8 *M1-T522 Flag2***	
**C-terminal_deletion_F**	GGCGCCGACTACAAGGAC
**M1_T522_R**	CGTCTCATCTGTTTGGCATACAC
**Mphosph8 *M1-S188 Flag2***	
**C-terminal_deletion_F**	GGCGCCGACTACAAGGAC
**M1_S188_R**	GCTCTCCAGCTCTGTCTTG
**Mphosph8 *E112-Q858 Flag2***	
**E112_Q858_F**	GAGAACAAAGCTAAAGCAGTCAG
**N-terminal_deletion_R**	CATGGTGGCAAGGGCGAA
**Mphosph8 *F59A;W80A;Y83A Flag2***	
**Mphosph8_sdm W80A F**	GGTATCATCTTCAGATGTATATCCTTTTGCTCGAACTTTATAAAGATTCTTACCTCC
**Mphosph8_sdm W80A R**	GGAGGTAAGAATCTTTATAAAGTTCGAGCAAAAGGATATACATCTGAAGATGATACC
**Mphosph8_sdm_F59A_F**	GAGGATGGGGAGGACGTTGCAGAGGTGGAGAGGATCC
**Mphosph8_sdm_F59A_R**	GGATCCTCTCCACCTCTGCAACGTCCTCCCCATCCTC
**Mphosph8_sdm_Y83_A_F**	GCTCCCAGGTATCATCTTCAGATGTTGCTCCTTTTGCTCGAACTTTATAAAGATTC
**Mphosph8_sdm_Y83A_R**	GAATCTTTATAAAGTTCGAGCAAAAGGAGCAACATCTGAAGATGATACCTGGGAGC
**Mphosph8 *∆ARD Flag2***	
**Mphosph8 ARD deletion F**	GAGCCACTTGGAGACACT
**Mphosph8 ARD deletion R**	CTCAACAAATTATTTGGAATCCTG
Cloning of ectopic Mpp8(mutant) vectors for expression into TasorFlag2 cells - Removal of flag2 tag	
**Mphosph8 *E112-Q858***	
**E112_Q858_F**	TAGAGGGCGAATTCGACC
**N-terminal_deletion_R**	CTGCAGCTGTACTCTGTATG
**Mphosph8 *M1-E729***	
**Flag2_Removal_F**	TAGAGGGCGAATTCGACC
**Flag2_Removal_1-729R**	TTCTGCAACTCTGGAAAGTG
**Mphosph8 *wt***	
**Flag2_Removal_F**	TAGAGGGCGAATTCGACC
**Flag2_Removal_Full-length R**	CTGCAGCTGTACTCTGTATG

Supplementary Table 8. Primers for qRT-PCR

Oligo name	Sequence
**Mphosph8 _CDS_exon3F**	GACAGTGATAGCGACCAGCAA
**Mphosph8 _CDS_exon3R**	CTCCAGCTCTGTCTTGAACTTGT
**Mphosph8_qPCR_F**	AGCTACCATCACCTGTGTTTG
**Mphosph8_qPCR_R**	CCTTTTCTGGTATTTTCCTTTGGG
**Mphosph8 3'UTR genomic F_1**	ACT ACC CTC ACC TAG GAC AGA T
**Mphosph8 3'UTR genomic R_1**	AAC GTT TTC GAC CAA TGG CA
**L1_ORF2_Bodak_F**	GGAGGGACATTTCATTCTCATCA
**L1_ORF2_Bodak_R**	GCTGCTCTTGTATTTGGAGCATAGA
**L1_A_Bodak_F**	GGATTCCACACGTGATCCTAA
**L1_A_Bodak_R**	TCCTCTATGAGCAGACCTGGA
**SINE_Bodak_F**	GTGGCGCACGCCTTTAATC
**SINE_Bodak_R**	GACAGGGTTTCTCTGTGTAG

Supplementary Table 9. Antibodies

Target	Host/Class [clone]	Supplier	Catalog number	Dilution WB	Dilution ChIP	Application
**MPP8**	Rabbit polyclonal	Proteintech	16796-1-AP	1:500	5 µl antibody to 300 µg chromatin	WB, ChIP
**Vinculin**	Mouse monoclonal [V284]	Sigma	SAB4200080	1:10000		WB
**FLAG**	Mouse monoclonal [M2]	Sigma	F3165	1:5000		WB
**ßACTIN**	Mouse monoclonal [AC-74]	Sigma	A2228	1:20000		WB
**Cas9**	Mouse monoclonal [7A9-3A3]	Cell Signaling	14697	1:1000		WB
**OsTIR1**	Rabbit polyclonal	MBL	PD048	1:1000		WB
**SETDB1**	Mouse monoclonal [5H6A12]	Abcam	Ab107225	1:500		WB
**H3K9me3**	Rabbit monoclonal [EPR16601]	Abcam	Ab176916		3 µl antibody to 50 µg chromatin	ChIP
**L1ORF1**	Rabbit monoclonal [EPR21844-108]	Abcam	Ab216324	1:1000		WB
**Tubulin**	Rabbit monoclonal [EPR13478]	Abcam	ab176560	1:5000		WB
**H3K4me3**	Rabbit monoclonal [C42D8]	Cell signaling	9751		2 µl antibody to 50 µg chromatin	ChIP
**H3K27ac**	Mouse monoclonal [MABI 0309]	Active motive	39685		5 µl antibody to 50 µg chromatin	ChIP
**Goat Anti-Rabbit IgG Antibody (H+L), Peroxidase**	Goat polyclonal	Vector Laboratories	PI-1000	1:10000		WB
**Horse Anti-Mouse IgG Antibody (H+L), Peroxidase**	Horse polyclonal	Vector Laboratories	PI-2000	1:10000		WB
**IRDye® 800CW Goat anti-Rabbit IgG**	Goat polyclonal	LI-COR Biosciences	925-32211	1:10000		WB
**IRDye® 680RD Goat anti-Mouse IgG**	Goat polyclonal	LI-COR Biosciences	926-68070	1:10000		WB

Supplementary Table 10. Primers used for ChIP-qPCR

Oligo name	Sequence
**Kcnq1ot1_genomic_F3**	GGACTAGCTGGCAAACCTTCA
**Kcnq1ot1_genomic_R3**	CCAGGCCAATCCAAAGCATTC
**L1Md_F2_chr2_primer1_ChIP_qPCR_F**	GGCAACTTAAAAAGGCTTAATTGGG
**L1Md_F2_chr2_primer1_ChIP_qPCR_R**	ACCAAGATGGCTTCTGTCGTT
**Srrm2_genomic_F2**	GCTCGAAGAGGTTCCAGGTC
**Srrm2_genomic_R2**	CTGGAGCGGATTTCTGGTGA
**Zfp617_primer1_ChIP_qPCR_F**	CAATATCCCCACACCTGGATT
**Zfp617_primer1_ChIP_qPCR_R**	ATCGAAATGATGAATGGATAGAACC
**Utp6_F**	TCTATGGCCTTACCCACTGC
**Utp6_R**	TGACACGTTTCTGCTTCCAG
**Pmp22_F**	AGCCACCATGCTCCTACTCT
**Pmp22_R**	GAAGAGCAACACTAGCACCG
