# Supplementary material for: MPP8 is essential for sustaining self-renewal of ground-state pluripotent stem cells
Source: Nat Commun. 2021 May 24;12:3034. doi: 10.1038/s41467-021-23308-4 (PMC8144423; doi:10.1038/s41467-021-23308-4)
Supplement: Supplementary file 3 — Reporting Summary [file 41467_2021_23308_MOESM3_ESM.pdf]

nature research

Corresponding author(s): Kristian Helin

Last updated by author(s): March 22, 2021

Reporting Summary

Nature Research wishes to improve the reproducibility of the work that we publish. This form provides structure for consistency and transparency in reporting. For further information on Nature Research policies, see our Editorial Policies and the Editorial Policy Checklist.

Statistics

For all statistical analyses, confirm that the following items are present in the figure legend, table legend, main text, or Methods section.

n/a	Confirmed	
☐	☒	The exact sample size (*n*) for each experimental group/condition, given as a discrete number and unit of measurement
☐	☒	A statement on whether measurements were taken from distinct samples or whether the same sample was measured repeatedly
☐	☒	The statistical test(s) used AND whether they are one- or two-sided<br>*Only common tests should be described solely by name; describe more complex techniques in the Methods section.*
☒	☐	A description of all covariates tested
☐	☒	A description of any assumptions or corrections, such as tests of normality and adjustment for multiple comparisons
☐	☒	A full description of the statistical parameters including central tendency (e.g. means) or other basic estimates (e.g. regression coefficient) AND variation (e.g. standard deviation) or associated estimates of uncertainty (e.g. confidence intervals)
☐	☒	For null hypothesis testing, the test statistic (e.g. *F*, *t*, *r*) with confidence intervals, effect sizes, degrees of freedom and *P* value noted<br>*Give P values as exact values whenever suitable.*
☒	☐	For Bayesian analysis, information on the choice of priors and Markov chain Monte Carlo settings
☒	☐	For hierarchical and complex designs, identification of the appropriate level for tests and full reporting of outcomes
☐	☒	Estimates of effect sizes (e.g. Cohen's *d*, Pearson's *r*), indicating how they were calculated

Our web collection on statistics for biologists contains articles on many of the points above.

Software and code

Policy information about availability of computer code

Data collection	Flow cytometry: BD Bioscience LSR II<br>Mass Spectrometry: Q Exactive UHMR Hybrid Quadrupole-Orbitrap Mass Spectrometer<br>ChIP-seq and RNA-seq: NextSeq 550
Data analysis	Flow cytometry:<br>BD FACSDIVA v.8.0.2<br>Flowjo v.9/v10<br>Mass Spectrometry:<br>Proteome Discoverer v2.2<br>Perseus v.1.6.14.0<br>ChIP-seq and RNA-seq:<br>bcl2fastq2 v.2.20.0<br>Trim Galore v.0.4.5<br>STAR v.2.5.3a/2.6.0a<br>epic2 v.0.0.41<br>IGV v.2.8.0<br>ngsplot v.2.63<br>Homer v.4.9<br>bedtools v.2.27.1<br>samtools v.1.10<br>R v.3.6.0<br>Statistics:

GraphPad Prism v.8.4.2
Western blot:
Image Studio Lite v.5.2.5

For manuscripts utilizing custom algorithms or software that are central to the research but not yet described in published literature, software must be made available to editors and reviewers. We strongly encourage code deposition in a community repository (e.g. GitHub). See the Nature Research guidelines for submitting code & software for further information.

Data

Policy information about availability of data

All manuscripts must include a data availability statement. This statement should provide the following information, where applicable:
- Accession codes, unique identifiers, or web links for publicly available datasets
- A list of figures that have associated raw data
- A description of any restrictions on data availability

The mass spectrometry data have been deposited to the ProteomeXchange Consortium via the PRIDE partner repository (PXD019345); ChIP-seq and RNA-seq data have been submitted to the Gene Expression Omnibus (GSE150926)

Field-specific reporting

Please select the one below that is the best fit for your research. If you are not sure, read the appropriate sections before making your selection.

☒ Life sciences ☐ Behavioural & social sciences ☐ Ecological, evolutionary & environmental sciences

For a reference copy of the document with all sections, see nature.com/documents/nr-reporting-summary-flat.pdf

Life sciences study design

All studies must disclose on these points even when the disclosure is negative.

Sample size	Sample sizes were chosen based on previous published literature in the field. Typically 2 or 3 independent experiments or independent biological samples were included, with some exceptions where only one independent experiment was performed. Sample sizes are indicated for all experiments. RNA-Seq analysis is based on two biological samples per condition, mass spectrometry analysis was based on two or three biological samples per condition. ChIP-Seq was typically performed once but validated using orthogonal approaches, e.g. MPP8 and FLAG ChIP-Seq for the presence of MPP8 (mutants) on chromatin and H3K4me3 and H3K27ac ChIP-Seq for the evaluation of changes in permissive chromatin. ChIP-seq was vigorously validated using ChIP qPCR analysis.
Data exclusions	No data exclusion was performed in this study
Replication	All attempts of replication were successful. The exact number of times each experiment was performed is stated in the corresponding figure legend.
Randomization	No randomization was performed in this study as it involves no group assignment but is based on objective molecular and cell biology methods
Blinding	No blinding was performed in this study as all data was obtained using objective, quantitative methods

Reporting for specific materials, systems and methods

We require information from authors about some types of materials, experimental systems and methods used in many studies. Here, indicate whether each material, system or method listed is relevant to your study. If you are not sure if a list item applies to your research, read the appropriate section before selecting a response.

Materials & experimental systems

n/a	Involved in the study	
☐	☒	Antibodies
☐	☒	Eukaryotic cell lines
☒	☐	Palaeontology and archaeology
☒	☐	Animals and other organisms
☒	☐	Human research participants
☒	☐	Clinical data
☒	☐	Dual use research of concern

Methods

n/a	Involved in the study	
☐	☒	ChIP-seq
☐	☒	Flow cytometry
☒	☐	MRI-based neuroimaging

Antibodies

Antibodies used	Primary antibodies:<br>Antibody, Supplier, Catalog Number, Host/Class [clone], Application, Dilution<br>anti-MPP8, Proteintech, 16796-1-AP, rabbit polyclonal, Western blot, 1:500<br>anti-MPP8, Proteintech, 16796-1-AP, rabbit polyclonal, ChIP, 5 µl antibody to 300 µg chromatin<br>anti-Vinculin, Sigma, SAB4200080, mouse monoclonal [V284], Western blot, 1:10000<br>anti-FLAG, Sigma, F3165, mouse monoclonal [M2], Western blot, 1:5000<br>anti-ßACTIN, Sigma, A2228, mouse monoclonal [AC-74], Western blot, 1:20000<br>anti-Cas9, Cell Signallig, 14697, mouse monoclonal [7A9-3A3], Western blot, 1:1000<br>anti-OsTIR1, MBL, PD048, rabbit polyclonal, Western blot, 1:1000<br>anti-SETDB1, Abcam, Ab107225, mouse monoclonal [5H6A12], Western blot, 1:500<br>anti-H3K9me3, Abcam, Ab176916, rabbit monoclonal [EPR16601], ChIP, 3 µl antibody to 50 µg chromatin<br>anti-L1ORF1, Abcam, Ab216324, rabbit monoclonal [EPR21844-108], Western blot, 1:1000<br>anti-Tubulin, Abcam, ab176560, rabbit monoclonal [EPR13478], Western blot, 1:5000<br>anti-H3K4me3, Cell signaling, 9751, Rabbit monoclonal [C42D8], ChIP, 2 µl antibody to 50 µg chromatin<br>anti-H3K27ac, Active motive, 39685, mouse monoclonal [MABI 0309], ChIP, 5 µl antibody to 50 µg chromatin<br><br>Secondary antibodies:<br>Antibody, Supplier, Catalog Number, Host/Class [clone], Application, Dilution<br>Goat Anti-Rabbit IgG Antibody (H+L) Peroxidase, Vector Laboratories, PI-1000, goat polyclonal, Western blot, 1:10000<br>Horse Anti-Mouse IgG Antibody (H+L) Peroxidase (Vector Laboratories, PI-2000), horse polyclonal, Western blot, 1:10000<br>IRDye® 800CW Goat anti-Rabbit IgG (LI-COR Biosciences, 925-32211), goat polyclonal, Western blot, 1:10000<br>IRDye® 680RD Goat anti-Mouse IgG (LI-COR Biosciences, 926-68070), goat polyclonal, Western blot, 1:10000
Validation	anti-MPP8: validated by the removal of the protein using the mAID/OsTIR1-mediated degron system (Supplementary Fig. 3a)<br>anti-Vinculin: Anti-Vinculin antibody, Mouse monoclonal has been used to probe blots in immunoblotting. (https://www.sigmaaldrich.com/catalog/product/sigma/sab4200080?lang=en&region=US)<br>anti-FLAG: validated by comparing the presence of the ectopically expressed or endogenously tagged MPP8 protein to wildtype cell lines (Supplementary Fig. 4b, 5f)<br>anti-ßACTIN: Monoclonal Anti-β-Actin antibody has been used in western blot (https://www.sigmaaldrich.com/catalog/product/sigma/a2228?lang=en&region=US)<br>anti-Cas9: validated by comparing the presence of the ectopically expressed protein to wildtype cell lines (Supplementary Fig. 1d)<br>anti-OsTIR1: validated by comparing the presence of the ectopically expressed protein to wildtype cell lines (Fig. 2b)<br>anti-SETDB1: validated by the knockout of the Setdb1 gene using a conditional Setdb1 KO cell line (Supplementary Fig. 9a)<br>anti-H3K9me3:<br>- validated using an ELISA-based approach against a modified histone peptide substrate library as described by Pedersen et al., 2014 (PMID:27266524)<br>- validated using H3K9me3-methyltransferase knockout cell lines (Fig. 6b)<br>anti-L1ORF1: Suitable for: WB, reacts with mouse (https://www.abcam.com/line-1-orf1p-antibody-epr21844-108-ab216324.html)<br>anti-Tubulin: Suitable for: WB (https://www.abcam.com/alpha-tubulin-antibody-epr13478b-loading-control-ab176560.html)<br>anti-H3K4me4: ChIP-validated, reactivity against mouse. Tri-Methyl-Histone H3 (Lys4) Antibody detects endogenous levels of histone H3 when tri-methylated on Lys4. This antibody shows some cross-reactivity with histone H3 that is di-methylated on Lys4, but does not cross-react with non-methylated or mono-methylated histone H3 Lys4. In addition, the antibody does not cross-react with methylated histone H3 Lys9, Lys27, Lys36 or methylated histone H4 Lys20 (https://www.cellsignal.com/products/primary-antibodies/tri-methyl-histone-h3-lys4-c42d8-rabbit-mab/9751)<br>-Anti-H3K27ac: ChIP-Seq validation was performed by Active Motif's Epigenetics Services. (https://www.activemotif.com/catalog/details/39685)

Eukaryotic cell lines

Policy information about cell lines

Cell line source(s)	E14-TG2a (129/Ola) mESCs were a kind gift from J. Brickman, RRID:CVCL_C320, commercial source: ATCC<br>HEK293FT cells were commercially bought from ATCC, RRID: CVCL_6911<br>NIH 3T3 mouse embryonic fibroblasts were kindly provided by J. Taipale, RRID:CVCL_0594<br>TCF2.2 (129B6F1) mESCs were derived by the transgenic core at UCPH (Martin Gonzalez et al., 2016)<br>NSCs were isolated in this laboratory as described in Mohammad et al. 2017<br>Trp53-/- MEFs were isolated in this laboratory as described in Aggar et al. 2009.
Authentication	No cell authentication was performed
Mycoplasma contamination	All the cells in this study were mycoplasma negative
Commonly misidentified lines<br>(See ICLAC register)	No commonly misidentified cell lines were used in this study.

ChIP-seq

Data deposition

☒ Confirm that both raw and final processed data have been deposited in a public database such as GEO.

☒ Confirm that you have deposited or provided access to graph files (e.g. BED files) for the called peaks.

Data access links<br>*May remain private before publication.*	ChIP-seq data have been deposited to the Gene Expression Omnibus under accession code GSE150926.
Files in database submission	ChIPSeq_IP_FLAG_wt_nothing_untreated.bw<br>ChIPSeq_IP_FLAG_Mpp8mAID_WT_IAA_16hrs.bw<br>ChIPSeq_IP_FLAG_Mpp8mAID_Cpart_IAA_16hrs.bw<br>ChIPSeq_IP_FLAG_Mpp8mAID_Npart_IAA_16hrs.bw<br>ChIPSeq_IP_MPP8_Mpp8mAID_nothing_untreated.bw<br>ChIPSeq_IP_MPP8_Mpp8mAID_nothing_IAA_16hrs.bw<br>ChIPSeq_IP_MPP8_Mpp8mAID_WT_IAA_16hrs.bw<br>ChIPSeq_IP_MPP8_Mpp8mAID_Cpart_IAA_16hrs.bw<br>ChIPSeq_IP_MPP8_Mpp8mAID_Npart_IAA_16hrs.bw<br>ChIPSeq_Input_Mpp8flag.bw<br>ChIPSeq_IP_FLAG_Mpp8flag.bw<br>ChIPSeq_IP_FLAG_Pphln1flag.bw<br>ChIPSeq_IP_FLAG_Tasorflag.bw<br>ChIPSeq_IP_FLAG_wt.bw<br>ChIPSeq_Input_Mpp8mAID_nothing_untreated_6hrs.bw<br>ChIPSeq_IP_H3K9me3_Mpp8mAID_nothing_untreated_6hrs.bw<br>ChIPSeq_Input_Mpp8mAID_nothing_IAA_6hrs.bw<br>ChIPSeq_IP_H3K9me3_Mpp8mAID_nothing_IAA_6hrs.bw<br>ChIPSeq_Input_Mpp8mAID_WT_untreated_6hrs.bw<br>ChIPSeq_IP_H3K9me3_Mpp8mAID_WT_untreated_6hrs.bw<br>ChIPSeq_Input_Mpp8mAID_WT_IAA_6hrs.bw<br>ChIPSeq_IP_H3K9me3_Mpp8mAID_WT_IAA_6hrs.bw<br>ChIPSeq_Input_Mpp8mAID_Npart_untreated_6hrs.bw<br>ChIPSeq_IP_H3K9me3_Mpp8mAID_Npart_untreated_6hrs.bw<br>ChIPSeq_Input_Mpp8mAID_Npart_IAA_6hrs.bw<br>ChIPSeq_IP_H3K9me3_Mpp8mAID_Npart_IAA_6hrs.bw<br>ChIPSeq_Input_Mpp8mAID_Cpart_untreated_6hrs.bw<br>ChIPSeq_IP_H3K9me3_Mpp8mAID_Cpart_untreated_6hrs.bw<br>ChIPSeq_Input_Mpp8mAID_Cpart_IAA_6hrs.bw<br>ChIPSeq_IP_H3K9me3_Mpp8mAID_Cpart_IAA_6hrs.bw<br>ChIPSeq_Input_Mpp8mAID_nothing_untreated_48hrs.bw<br>ChIPSeq_IP_H3K9me3_Mpp8mAID_nothing_untreated_48hrs.bw<br>ChIPSeq_Input_Mpp8mAID_nothing_IAA_48hrs.bw<br>ChIPSeq_IP_H3K9me3_Mpp8mAID_nothing_IAA_48hrs.bw<br>ChIPSeq_Input_Mpp8mAID_WT_untreated_48hrs.bw<br>ChIPSeq_IP_H3K9me3_Mpp8mAID_WT_untreated_48hrs.bw<br>ChIPSeq_Input_Mpp8mAID_WT_IAA_48hrs.bw<br>ChIPSeq_IP_H3K9me3_Mpp8mAID_WT_IAA_48hrs.bw<br>ChIPSeq_Input_Mpp8mAID_Npart_untreated_48hrs.bw<br>ChIPSeq_IP_H3K9me3_Mpp8mAID_Npart_untreated_48hrs.bw<br>ChIPSeq_Input_Mpp8mAID_Npart_IAA_48hrs.bw<br>ChIPSeq_IP_H3K9me3_Mpp8mAID_Npart_IAA_48hrs.bw<br>ChIPSeq_Input_Mpp8mAID_Cpart_untreated_48hrs.bw<br>ChIPSeq_IP_H3K9me3_Mpp8mAID_Cpart_untreated_48hrs.bw<br>ChIPSeq_Input_Mpp8mAID_Cpart_IAA_48hrs.bw<br>ChIPSeq_IP_H3K9me3_Mpp8mAID_Cpart_IAA_48hrs.bw<br>MPP8ChIPseq_peaks_epic2.bed<br>ChIPSeq_Input_wt_nothing_untreated_R1.fastq.gz<br>ChIPSeq_IP_FLAG_wt_nothing_untreated_R1.fastq.gz<br>ChIPSeq_Input_Mpp8mAID_WT_IAA_16hrs_R1.fastq.gz<br>ChIPSeq_IP_FLAG_Mpp8mAID_WT_IAA_16hrs_R1.fastq.gz<br>ChIPSeq_Input_Mpp8mAID_Cpart_IAA_16hrs_R1.fastq.gz<br>ChIPSeq_IP_FLAG_Mpp8mAID_Cpart_IAA_16hrs_R1.fastq.gz<br>ChIPSeq_Input_Mpp8mAID_Npart_IAA_16hrs_R1.fastq.gz<br>ChIPSeq_IP_FLAG_Mpp8mAID_Npart_IAA_16hrs_R1.fastq.gz<br>ChIPSeq_IP_MPP8_Mpp8mAID_nothing_untreated_R1.fastq.gz<br>ChIPSeq_IP_MPP8_Mpp8mAID_nothing_IAA_16hrs_R1.fastq.gz

ChIPSeq_IP_MPP8_Mpp8mAID_WT_IAA_16hrs_R1.fastq.gz
ChIPSeq_IP_MPP8_Mpp8mAID_Cpart_IAA_16hrs_R1.fastq.gz
ChIPSeq_IP_MPP8_Mpp8mAID_Npart_IAA_16hrs_R1.fastq.gz
ChIPSeq_Input_wt_nothing_untreated_R2.fastq.gz
ChIPSeq_IP_FLAG_wt_nothing_untreated_R2.fastq.gz
ChIPSeq_Input_Mpp8mAID_WT_IAA_16hrs_R2.fastq.gz
ChIPSeq_IP_FLAG_Mpp8mAID_WT_IAA_16hrs_R2.fastq.gz
ChIPSeq_Input_Mpp8mAID_Cpart_IAA_16hrs_R2.fastq.gz
ChIPSeq_IP_FLAG_Mpp8mAID_Cpart_IAA_16hrs_R2.fastq.gz
ChIPSeq_Input_Mpp8mAID_Npart_IAA_16hrs_R2.fastq.gz
ChIPSeq_IP_FLAG_Mpp8mAID_Npart_IAA_16hrs_R2.fastq.gz
ChIPSeq_IP_MPP8_Mpp8mAID_nothing_untreated_R2.fastq.gz
ChIPSeq_IP_MPP8_Mpp8mAID_nothing_IAA_16hrs_R2.fastq.gz
ChIPSeq_IP_MPP8_Mpp8mAID_WT_IAA_16hrs_R2.fastq.gz
ChIPSeq_IP_MPP8_Mpp8mAID_Cpart_IAA_16hrs_R2.fastq.gz
ChIPSeq_IP_MPP8_Mpp8mAID_Npart_IAA_16hrs_R2.fastq.gz
ChIPSeq_Input_Mpp8flag_R1.fastq.gz
ChIPSeq_IP_FLAG_Mpp8flag_R1.fastq.gz
ChIPSeq_IP_FLAG_Pphln1flag_R1.fastq.gz
ChIPSeq_IP_FLAG_Tasorflag_R1.fastq.gz
ChIPSeq_IP_FLAG_wt_R1.fastq.gz
ChIPSeq_Input_Mpp8flag_R2.fastq.gz
ChIPSeq_IP_FLAG_Mpp8flag_R2.fastq.gz
ChIPSeq_IP_FLAG_Pphln1flag_R2.fastq.gz
ChIPSeq_IP_FLAG_Tasorflag_R2.fastq.gz
ChIPSeq_IP_FLAG_wt_R2.fastq.gz
ChIPSeq_Input_Mpp8mAID_nothing_untreated_6hrs_R1.fastq.gz
ChIPSeq_IP_H3K9me3_Mpp8mAID_nothing_untreated_6hrs_R1.fastq.gz
ChIPSeq_Input_Mpp8mAID_nothing_IAA_6hrs_R1.fastq.gz
ChIPSeq_IP_H3K9me3_Mpp8mAID_nothing_IAA_6hrs_R1.fastq.gz
ChIPSeq_Input_Mpp8mAID_WT_untreated_6hrs_R1.fastq.gz
ChIPSeq_IP_H3K9me3_Mpp8mAID_WT_untreated_6hrs_R1.fastq.gz
ChIPSeq_Input_Mpp8mAID_WT_IAA_6hrs_R1.fastq.gz
ChIPSeq_IP_H3K9me3_Mpp8mAID_WT_IAA_6hrs_R1.fastq.gz
ChIPSeq_Input_Mpp8mAID_Npart_untreated_6hr_R1.fastq.gz
ChIPSeq_IP_H3K9me3_Mpp8mAID_Npart_untreated_6hrs_R1.fastq.gz
ChIPSeq_Input_Mpp8mAID_Npart_IAA_6hrs_R1.fastq.gz
ChIPSeq_IP_H3K9me3_Mpp8mAID_Npart_IAA_6hrs_R1.fastq.gz
ChIPSeq_Input_Mpp8mAID_Cpart_untreated_6hrs_R1.fastq.gz
ChIPSeq_IP_H3K9me3_Mpp8mAID_Cpart_untreated_6hrs_R1.fastq.gz
ChIPSeq_Input_Mpp8mAID_Cpart_IAA_6hrs_R1.fastq.gz
ChIPSeq_IP_H3K9me3_Mpp8mAID_Cpart_IAA_6hrs_R1.fastq.gz
ChIPSeq_Input_Mpp8mAID_nothing_untreated_6hrs_R2.fastq.gz
ChIPSeq_IP_H3K9me3_Mpp8mAID_nothing_untreated_6hrs_R2.fastq.gz
ChIPSeq_Input_Mpp8mAID_nothing_IAA_6hrs_R2.fastq.gz
ChIPSeq_IP_H3K9me3_Mpp8mAID_nothing_IAA_6hrs_R2.fastq.gz
ChIPSeq_Input_Mpp8mAID_WT_untreated_6hrs_R2.fastq.gz
ChIPSeq_IP_H3K9me3_Mpp8mAID_WT_untreated_6hrs_R2.fastq.gz
ChIPSeq_Input_Mpp8mAID_WT_IAA_6hrs_R2.fastq.gz
ChIPSeq_IP_H3K9me3_Mpp8mAID_WT_IAA_6hrs_R2.fastq.gz
ChIPSeq_Input_Mpp8mAID_Npart_untreated_6hr_R2.fastq.gz
ChIPSeq_IP_H3K9me3_Mpp8mAID_Npart_untreated_6hrs_R2.fastq.gz
ChIPSeq_Input_Mpp8mAID_Npart_IAA_6hrs_R2.fastq.gz
ChIPSeq_IP_H3K9me3_Mpp8mAID_Npart_IAA_6hrs_R2.fastq.gz
ChIPSeq_Input_Mpp8mAID_Cpart_untreated_6hrs_R2.fastq.gz
ChIPSeq_IP_H3K9me3_Mpp8mAID_Cpart_untreated_6hrs_R2.fastq.gz
ChIPSeq_Input_Mpp8mAID_Cpart_IAA_6hrs_R2.fastq.gz
ChIPSeq_IP_H3K9me3_Mpp8mAID_Cpart_IAA_6hrs_R2.fastq.gz
ChIPSeq_Input_Mpp8mAID_nothing_untreated_48hrs_R1.fastq.gz
ChIPSeq_IP_H3K9me3_Mpp8mAID_nothing_untreated_48hrs_R1.fastq.gz
ChIPSeq_Input_Mpp8mAID_nothing_IAA_48hrs_R1.fastq.gz
ChIPSeq_IP_H3K9me3_Mpp8mAID_nothing_IAA_48hrs_R1.fastq.gz
ChIPSeq_Input_Mpp8mAID_WT_untreated_48hrs_R1.fastq.gz
ChIPSeq_IP_H3K9me3_Mpp8mAID_WT_untreated_48hrs_R1.fastq.gz
ChIPSeq_Input_Mpp8mAID_WT_IAA_48hrs_R1.fastq.gz
ChIPSeq_IP_H3K9me3_Mpp8mAID_WT_IAA_48hrs_R1.fastq.gz
ChIPSeq_Input_Mpp8mAID_Npart_untreated_48hr_R1.fastq.gz
ChIPSeq_IP_H3K9me3_Mpp8mAID_Npart_untreated_48hrs_R1.fastq.gz

	ChIPSeq_Input_Mpp8mAID_Npart_IAA_48hrs_R1.fastq.gz<br>ChIPSeq_IP_H3K9me3_Mpp8mAID_Npart_IAA_48hrs_R1.fastq.gz<br>ChIPSeq_Input_Mpp8mAID_Cpart_untreated_48hrs_R1.fastq.gz<br>ChIPSeq_IP_H3K9me3_Mpp8mAID_Cpart_untreated_48hrs_R1.fastq.gz<br>ChIPSeq_Input_Mpp8mAID_Cpart_IAA_48hrs_R1.fastq.gz<br>ChIPSeq_IP_H3K9me3_Mpp8mAID_Cpart_IAA_48hrs_R1.fastq.gz<br>ChIPSeq_Input_Mpp8mAID_nothing_untreated_48hrs_R2.fastq.gz<br>ChIPSeq_IP_H3K9me3_Mpp8mAID_nothing_untreated_48hrs_R2.fastq.gz<br>ChIPSeq_Input_Mpp8mAID_nothing_IAA_48hrs_R2.fastq.gz<br>ChIPSeq_IP_H3K9me3_Mpp8mAID_nothing_IAA_48hrs_R2.fastq.gz<br>ChIPSeq_Input_Mpp8mAID_WT_untreated_48hrs_R2.fastq.gz<br>ChIPSeq_IP_H3K9me3_Mpp8mAID_WT_untreated_48hrs_R2.fastq.gz<br>ChIPSeq_Input_Mpp8mAID_WT_IAA_48hrs_R2.fastq.gz<br>ChIPSeq_IP_H3K9me3_Mpp8mAID_WT_IAA_48hrs_R2.fastq.gz<br>ChIPSeq_Input_Mpp8mAID_Npart_untreated_48hr_R2.fastq.gz<br>ChIPSeq_IP_H3K9me3_Mpp8mAID_Npart_untreated_48hrs_R2.fastq.gz<br>ChIPSeq_Input_Mpp8mAID_Npart_IAA_48hrs_R2.fastq.gz<br>ChIPSeq_IP_H3K9me3_Mpp8mAID_Npart_IAA_48hrs_R2.fastq.gz<br>ChIPSeq_Input_Mpp8mAID_Cpart_untreated_48hrs_R2.fastq.gz<br>ChIPSeq_IP_H3K9me3_Mpp8mAID_Cpart_untreated_48hrs_R2.fastq.gz<br>ChIPSeq_Input_Mpp8mAID_Cpart_IAA_48hrs_R2.fastq.gz<br>ChIPSeq_IP_H3K9me3_Mpp8mAID_Cpart_IAA_48hrs_R2.fastq.gz
Genome browser session (e.g. UCSC)	All bigwigs available to download for browsing in genome browser of choice.

Methodology

Replicates	MPP8 ChIP-Seq in MPP8mAID; OsTIR1 cells and cells additionally expressing MPP8 mutants was conducted in one biological experiment, an orthogonal approach in which the same cell lines were ChIP-seqed using a FLAG antibody was conducted in one other biological experiment. FLAG ChIP-seq on other endogenous-tagged HUSH complex members (TASOR, PPHLN1) was conducted once. H3K9me3 ChIP-Seq in MPP8mAID; OsTIR1 cells and cells additionally expressing MPP8 mutants was conducted in one biological experiment at two different timepoints after MPP8 depletion. Peaks were extensively validated using ChIP-qPCR.
Sequencing depth	ChIP-seq samples were paired-ended for all samples, 75 bp was the read length. On average each ChIP-seq sample contained 63M reads with min 20M and max 139M reads.
Antibodies	MPP8 (Proteintech,16796-1-AP), FLAG (Affinity gel, Sigma Aldrich, A2220) and H3K9me3 (Abcam, Ab176916) were used for ChIP-seq.
Peak calling parameters	Reads were first trimmed using Trim Galore (Version 0.4.5) using --illumina and default parameters. Mapping was done with STAR (Version 2.5.3a) allowing multimappers (--winAnchorMultimaNmax 100 --outFilterMultimapNmax 100 --outFilterMismatchNmax 3) to mm10 assembly. Peaks were called for using epic2 peak caller (version 0.0.41) using the epic2-df function. Peaks were subsequently filtered to fulfil wildtype read number requirements (>= 100 && <=5000) and for peaks that are overrepresented in the wildtype condition (FC_WT > 1) yielding 55 high confidence peaks.
Data quality	FDR from epic2 peak calling was set at default 5% and only peaks that were overrepresented in WT over KO conditions (FC_WT > 1)
Software	bcl2fastq2 (Version 2.20.0), Trim Galore (Version 0.4.5) , STAR (Version 2.5.3a and 2.6.0a) , epic2 peak caller (version 0.0.41), IGV (version 2.8.0), ngsplot (Version 2.63) , homer (Version 4.9), bedtools (version 2.27.1), samtools (version 1.10), R (version R-3.6.0);]

Flow Cytometry

Plots

Confirm that:

- [x] The axis labels state the marker and fluorochrome used (e.g. CD4-FITC).
- [x] The axis scales are clearly visible. Include numbers along axes only for bottom left plot of group (a 'group' is an analysis of identical markers).
- [x] All plots are contour plots with outliers or pseudocolor plots.
- [x] A numerical value for number of cells or percentage (with statistics) is provided.

Methodology

Sample preparation	No special sample preparation was necessary, live cells were sorted with no staining involved.
Instrument	BD LSR II
Software	BD FACSDiva was used for the acquisition analysis and FlowJO for further processing.

Cell population abundance	10000 cells were acquired per sample in competition-based proliferation assays and cell cycle analysis.
Gating strategy	Cells were gated for live/dead and doublet exclusion using FSC and SSC channels. Then cells were further gated for the presence of GFP (Competition-based proliferation assays, knockin of endogenous 2xFlag tags at Mphosph8, Tasor and Pphln1 loci, generation of Mphosph8 knockout NIH 3T3 cells) or for GFP/BFP double-positive cells (knockin of mAID-T2A-BFP). Events passing above gating strategy were classified as positive or negative based on SSC and GFP channel signals. For the presence of GFP or BFP, non-transfected/transduced cells were used as gating controls.

☒ Tick this box to confirm that a figure exemplifying the gating strategy is provided in the Supplementary Information.
