# Supplementary material for: MPP8 is essential for sustaining self-renewal of ground-state pluripotent stem cells
Source: Nat Commun. 2021 May 24;12:3034. doi: 10.1038/s41467-021-23308-4 (PMC8144423; doi:10.1038/s41467-021-23308-4)
Supplement: Supplementary file 4 — Description of Additional Supplementary Files [file 41467_2021_23308_MOESM4_ESM.pdf]

Description of Additional Supplementary Files

Supplementary Data 1: sgRNA sequences, raw counts of sgRNAs from the epi library CRISPR/Cas9 screen and identified hits

Supplementary Data 2: Differentially regulated transcripts obtained from RNA-seq

Supplementary Data 3: Protein abundances obtained from mass spectrometry using Proteome Discoverer software and statistical analysis performed using Perseus software

Supplementary Data 4: Genomic coordinates and gene annotations of MPP8 binding sites
